# Cluster Anions of Hydrated Polycyclic Aromatic Hydrocarbons: “Magic” Water Tetramer

**DOI:** 10.1021/acs.jpca.6c00211

**Published:** 2026-03-30

**Authors:** Jozef Ďurana, Barbora Kocábková, Andrij Pysanenko, Eva Pluhařová, Juraj Fedor, Michal Fárník

**Affiliations:** † J. Heyrovský Institute of Physical Chemistry, v.v.i., 86875 Czech Academy of Sciences, Dolejškova 2155/3, 182 23 Prague, Czech Republic; ‡ University of Chemistry and Technology Prague, Technická 5, 166 28 Prague 6, Czech Republic

## Abstract

The PAH-water (PAH = polycyclic aromatic hydrocarbon) interactions occur in interstellar ice grains and in atmospheres of planets and can serve as models for the interaction between water and graphite-like structures. We investigated clusters of several PAHs (naphthalene, cyanonaphthalene, phenanthrene, and anthracene) with water in a molecular beam experiment supported by theoretical calculations. The mass spectra of the negatively charged [(PAH)_*m*_(H_2_O)_*n*_]^−^ ions show strong “magic peaks” for *n* = 4. At the same time, it is well-known that the bare water tetramer anion (H_2_O)_4_^–^ is quite elusive. We argue that the observed magic peaks originate from the higher stability of the neutral water tetramer that is attached to the charged PAH cluster. Our calculations of the structure and energetics of the neutral clusters (Np)_*m*_(H_2_O)_*n*_ with *m* = 1 and 2 and *n* = 1–9 (Np = naphthalene) confirm this conclusion. They reveal another “magic” number *n* = 8 that is also seen experimentally. These findings advance our understanding of PAH-water interaction at a molecular level and provide preferred structural patterns for medium-size PAH-water clusters, which have implications for astrochemistry.

## Graphical abstract

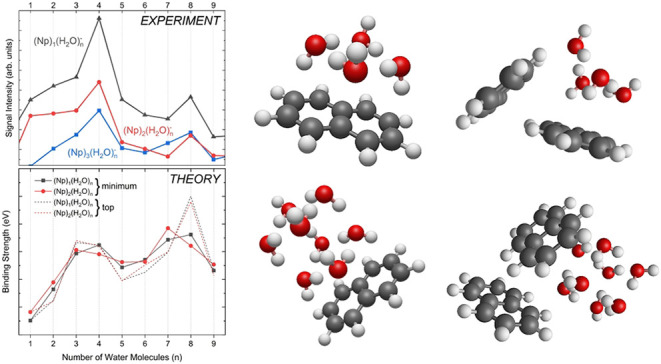

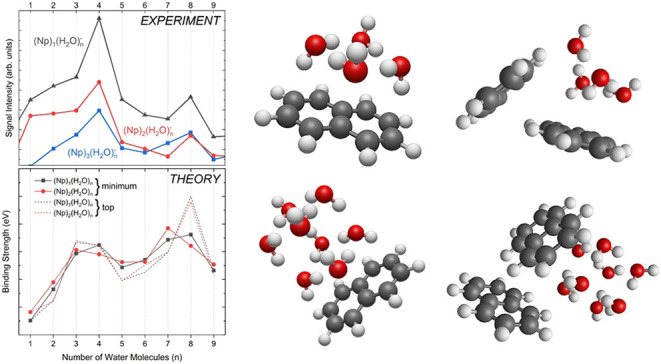

## Introduction

The water monomer cannot form a stable bound anion. A quasibound gas-phase H_2_O^–^ was detected,^1^ but it is unstable in geometries close to the equilibrium structure of the water molecule.^2,3^ Aggregated water molecules can cooperatively support a bound state for excess electrons, and negatively charged water clusters have been observed in many experiments.^4–12^ Clusters (H_2_O)_*n*_^–^ with *n* ≥ 11 can be easily produced in discharged supersonic expansions^4,5^ and/or in electron attachment experiments in a cross beam arrangement.^8,12^ In the small size regime, curious patterns occur in the mass spectra, with enhanced intensities (“magic numbers”) for *n* = 2, 6, and 7.^10,13^ Interestingly, the water tetramer (H_2_O)_4_^–^ was always missing in the spectra until Johnson et al. prepared it by an argon-mediated condensation and characterized it spectroscopically.^10^ Here, we show that, contrary to pure water anions, the tetramer appears as a strong “magic peak” in the negative ion mass spectra when attached to polycyclic aromatic hydrocarbons (PAHs). We argue that its higher abundance is due to the stability of the corresponding neutral cluster attached to the charged PAH.

PAH molecules themselves exhibit nontrivial behavior with respect to electron attachment due to the negatively charged π cloud of electrons on the aromatic ring. The electron affinity (EA) of benzene and naphthalene has a negative value of −1.12 eV^14^ and −0.20 eV,^15^ respectively. Thus, only short-lived transient negative ion (TNI) resonances can be formed from benzene and naphthalene. However, a solvation with other molecules can stabilize TNI.^16–21^ In larger PAHs, the π-orbital system expands and an excess electron can delocalize over the molecular framework, resulting in a positive EA. For anthracene and pyrene, EAs are 0.54 eV^22–24^ and 0.41 eV,^25^ respectively. The EA also depends on the molecular structure. Anthracene and phenanthrene both consist of three fused benzene rings C_14_H_10_, but the former is a linear molecule, while the latter is L-shaped. Anthracene has a positive EA, phenanthrene has a value close to zero^26–28^ with the most recent value of EA ≤ 0.025 eV.^29,30^ The anionic Np-water and other PAH-water complexes have been investigated by photoelectron spectroscopy.^15,31^ Several hydrated PAH anions were also characterized by infrared action spectroscopy accompanied by theoretical calculations.^32–34^

The heterogeneous PAH-water complexes are of interest in fundamental research as well as in various areas of applied chemistry. PAHs are abundant in interstellar space^35,36^ and can be found condensed in icy grain mantles.^37^ Thus, PAH-water complexes play an important role in the chemistry of cold molecular clouds,^35^ and understanding the dynamics of these complexes in interaction with electrons is essential for astrochemistry. The condensation of water and other molecules on PAHs can generate aerosol particles in planetary atmospheres, including our Earth, where PAHs are emitted as a result of various combustion processes. Such aerosols influence atmospheric chemistry and lead to cloud formation that changes the climate.^38,39^ The PAH-water clusters may also serve as models for understanding the interaction between water and graphite-like structures, which occurs in a variety of applications, including water purification, hydrogen production, etc.

The astrochemical interest motivated the spectroscopic characterization of neutral PAH-water complexes.^40–43^ IR spectra of naphthalene-water clusters with 1–3 water molecules were also recorded in helium nanodroplets.^44^ Spectroscopy, together with theoretical calculations^45–47^ suggested that water clusters tend to maximize the number of intracluster hydrogen bonds and form cyclic networks that are attached to PAHs by OH···π bonds. This general pattern has already been proposed in the pioneering work of Zwier’s group^48^ for benzene-water clusters based on multiphoton ionization mass spectrometry. Cluster cations of naphthalene-water complexes were investigated by IR photodissociation spectroscopy^49–51^ and by mass spectrometry after tunable vacuum ultraviolet (VUV) photoionization.^52^ The accompanying *ab initio* calculations suggested that the structure of the water subclusters attached to naphthalene was similar to that found in the isolated water clusters, and a similar study of anthracene suggested water confinement between two anthracenes in the case of four water molecules.^53,54^

Here, we focus on hydrated complexes of naphthalene (Np), 1-cyanonaphthalene (CNNp), anthracene (An), and phenanthrene (Ph). These PAHs were chosen to study the effect of size, structure, and EA. Np (C_10_H_8_) has an EA < 0, while in CNNp (C_10_H_7_CN), the CN group is responsible for its relatively large positive EA. An and Ph have the same composition C_10_H_14_ but differ in structure and EA, as explained above. The electron affinities are summarized in Table 1. An interesting aspect of the present study is the detection of relatively abundant [(PAH)_*m*_(H_2_O)_*n*_]^−^ anions for all investigated PAH molecules despite the very low or even negative electron affinities of water, Np and Ph. The most striking is the strong magic peak observed for *n* = 4, since the pure water tetramer anion (H_2_O)_4_^–^ itself is quite elusive, as mentioned above. We argue that the higher abundance of the anions [(PAH)_*m*_(H_2_O)_4_]^−^ originates from a higher stability of the neutral precursor (PAH)_*m*_(H_2_O)_4_ that is formed in supersonic expansion by sequential addition of water molecules to a PAH cluster, and after attachment of an electron, the charge resides in the PAH moiety.

**Table 1:** Electron Affinities (EA) of Naphthalene (Np), 1-Cyanonaphthalene (CNNp), Phenanthrene (Ph), Anthracene (An), and Water Molecules

molecule	Np	CNNp	Ph	An	H_2_O
EA (eV)	–0.20 ^15^	0.68 ^55^	≤0.025 ^29,30^	0.54 ^22–24^	<0 ^2,3^

## Methods

### Experimental Section

Our cluster beam (CLUB) apparatus was described previously.^56,57^ Only a brief description of the experiment and the parameters specific to the present study are given below. The clusters were produced in supersonic expansions of PAH vapors with buffer gas Ar at 1.5 bar. Water molecules were added to the buffer gas using Pergo humidifier (Elemental Scientific) described in our earlier study.^58^ Water molecules permeate through a membrane in the Nafion tubing, humidifying the argon gas flowing through the tubing. Unlike in our previous works,^58–62^ the Nafion membrane was not submerged in liquid water. Placing the tube close above the water sheet so that only the water vapor could permeate through resulted in a lower degree of humidification, where we can observe the magic numbers in the present mass spectra. With a higher water content, the magic numbers are less pronounced due to a higher abundance of the clusters with more water molecules. This is illustrated in Supporting Information (SI), Figure S5, which is showing CNNp spectra under low and high hydration conditions. Different reservoir temperatures *T*_R_ were used to evaporate the PAH samples. The nozzle temperature *T*_N_ was higher than *T*_R_ to prevent condensation and nozzle clogging. Table 2 summarizes the cluster source parameters.

**Table 2:** Experimental Conditions for Cluster Generation: Reservoir Temperature *T*_R_, Nozzle Temperature *T*_N_, Expansion Pressure *P*_0_ ^a^

PAH	*T*_R_ (°C)	*T*_N_ (°C)	P_0_ (bar)
naphthalene	82	96	1.5
1-cyanonaphthalene	150	160	1.5
anthracene	185	195	1.5
phenanthrene	195	205	1.5

a Conical nozzle parameters: diameter 90 μm, length 2 mm, full opening angle 30 deg.

After passing through a 0.8 mm skimmer, the clusters flew through several differentially pumped vacuum chambers on a flight path of ≈1.5 m (≈2.5 ms). Then, they were negatively charged with a cross beam of low energy electrons at *E*_e_ ≈ 2 eV. This value corresponds to the lowest well-defined electron energy of our electron gun, which was originally designed for higher electron energies of the order of tens of eV. We have shown in our previous studies that the electron beam with a nominal energy of 2 eV exhibits a relatively broad energy distribution also containing electrons with near-zero energies. The presence of 0 eV electrons in our beam was demonstrated by measuring electron attachment to SF_6_ and pure water clusters that exhibited a strong zero-energy resonance.^8^ We observed SF_6_^–^ and (H_2_O)_*n*_^–^ ions formed at nominal energies of *E*_e_ ≈ 2 eV. The electron attachment to water and the present PAH molecules occurs with near zero energy electrons^8,29,63^ (dissociative processes occur at energies significantly higher than 2 eV). Therefore, we argue that mainly slow electrons with near zero energies contribute to our [(PAH)_*m*_(H_2_O)_*n*_]^−^ signals after the electron attachment.

The negative ion mass spectra were measured with a reflectron time-of-flight mass spectrometer (RTOF). The first implementation of the RTOF on the CLUB apparatus in the negative ion detection mode was described elsewhere.^58,64^ In the present study, the electron beam was pulsed at 10 kHz, the ionizing pulse width was 3 μs. The ions were extracted after a delay of 0.2 μs with the extraction voltage pulse ±2 kV applied on the extractor/repeller plates. The extraction pulse width was 2 μs. The extracted ions were accelerated to 8 kV into the time-of-flight region, and in the end they were detected by the microchannel plate detector at 2.36 kV and mass spectra were recorded.

### Theoretical Calculations

The different structures of (Np)_1,2_(H_2_O)_1–9_ and (H_2_O)_1–10_ were optimized at the ωB97XD/aug-cc-pvdz level of theory^65,66^ in Gaussian 16 program package.^67^ The initial guesses of the geometries of various isomers of (Np)_1,2_(H_2_O)_1–9_ were based on analogy with pyrene,^68^ coronene,^47^ anthracene^53^ or prepared by placing water clusters next to Np_1_ or Np_2_ in different ways. The initial geometries of the pure water clusters were taken from the database.^69^ For the resulting structures, we performed a frequency analysis to confirm the local minima. To check the sensitivity of the results to the size of the basis set, we performed additional single point calculations employing the aug-cc-pvtz basis set for (Np)_1_(H_2_O)_1–9_ and the pure water clusters.

The obtained structures are visualized in Supporting Information (SI) Figures S6–S15 together with the relative energies of the isomers including the zero-point vibrational energy. To describe the stability of the clusters, we calculated two types of binding strength. The incremental one is related to the loss of a single water molecule: (Np)_*m*_(H_2_O)_*n*_ → (Np)_*m*_(H_2_O)_*n*−1_ + H_2_O. The absolute binding strength takes monomers as a reference: (Np)_*m*_(H_2_O)_*n*_ → *m* × Np + *n* × H_2_O. The energy differences are evaluated for the lowest energy isomers for each cluster size. Just in specific cases labeled as “on top”, the corresponding “on top” structures were used (see SI). Thus, the incremental binding strength characterizes the dissociation of any water molecule and subsequent rearrangement of the resulting smaller cluster. All energy differences include the zero-point vibrational energy calculated using the aug-cc-pvdz basis set.

## Results

### Measured Mass Spectra

The mass spectra of the [(PAH)_*m*_(H_2_O)_*n*_]^−^ negative cluster ions are shown in Figure 1. They correspond to the attachment of slow electrons with a nominal energy of ≈2 eV as explained in the Methods. The hydrated naphthalene spectrum in Figure 1a shows the series [(Np)_*m*_(H_2_O)_*n*_]^−^ (for each *m* the peaks corresponding to the series are connected by lines of different colors). As described above, the monomer anion (Np)_1_^–^ is an unstable resonance,^14,70^ and thus it is not present in the spectrum. The first clearly discernible ion is the Np anion with two water molecules [(Np)_1_(H_2_O)_2_]^−^ (red triangle). The abundance of cluster anion [(Np)_1_(H_2_O)_*n*_]^−^ rapidly increases with hydration to *n* = 4, then it drops dramatically for *n* ≥ 5. There is a second weaker maximum at *n* = 8. The naphthalene dimer anion (Np)_2_^–^ is already stable,^15,17,18,31^ however, it cannot be formed by an attachment to the neutral dimer.^21^ In the present spectrum, the first dimer peak is the one solvated with one water molecule [(Np)_2_(H_2_O)_1_]^−^ (blue triangle). The [(Np)_2_(H_2_O)_*n*_]^−^ series reaches its maximum again with *n* = 4 water molecules. The subsequent abrupt drop in peak intensities is interrupted by another maximum at *n* = 8. The bare anions (Np)_*m*_^–^ are observed from *m* ≥ 3. The corresponding [(Np)_*m*_(H_2_O)_*n*_]^−^ series for *m* = 3 and 4 show a similar behavior with a strong maximum at *n* = 4 and a weaker one at *n* = 8. Further series [(Np)_*m*_(H_2_O)_*n*_]^−^ for *m* ≥ 5 exhibit still maxima at *n* = 4 (shown only for *m* = 5 series) but they are not as pronounced. The *n* = 4 maxima are labeled in Figure 1 and correspond to the so-called “magic numbers”, as they exhibit a significantly higher abundance relative to the neighboring mass peaks.

**Figure 1:**
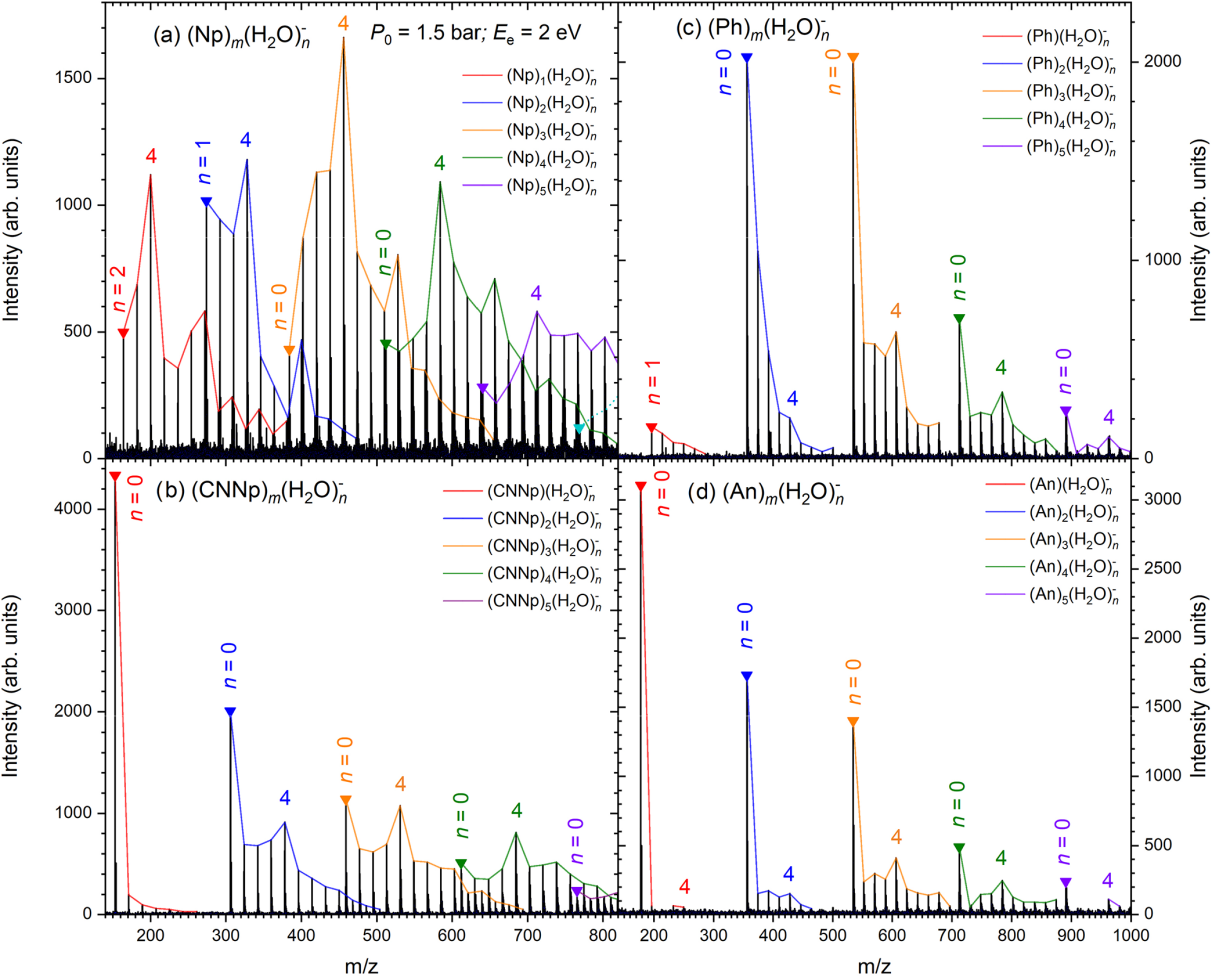
Mass spectra of the [(PAH)_*m*_(H_2_O)_*n*_]^−^ negative cluster ions measured for PAH = Np (a), CNNp (b), Ph (c), and An (d). The peaks of individual [(PAH)_*m*_(H_2_O)_*n*_]^−^ series with given *m* are connected by the colored lines; the first peak of each series is denoted by the triangle of the corresponding color and labeled by the corresponding number of water molecules *n*. Magic peaks *n* = 4 in each series are labeled.

Figure 1b shows the mass spectrum of hydrated cyanonaphthalene [(CNNp)_*m*_(H_2_O)_*n*_]^−^. The monomer (CNNp)_1_^–^ peak is the most abundant. The hydrated anion series [(CNNp)_*m*_(H_2_O)_*n*_]^−^ are present and clear magic peaks are observed at *n* = 4 for *m* = 2–4. For phenanthrene, the monomer anion (Ph)_1_^–^ is not present in the spectrum, Figure 1c, presumably due to the extremely low electron affinity.^29,30^ The first peak in the spectrum above the noise limit corresponds to the hydrated anion [(Ph)_1_(H_2_O)_1_]^−^. The pure cluster anions (Ph)_*m*_^–^ with *m* ≥ 2 are abundant. The hydrated cluster anion series [(Ph)_*m*_(H_2_O)_*n*_]^−^ again exhibit the *n* = 4 magic number for *m* = 3–5 (there is an indication of slightly increased intensity at *n* = 4 even for *m* = 1 and 2). Anthracene has a relatively high electron affinity; therefore, the pure (An)_*m*_^–^ series starts already with a strong monomer peak (An)_1_^–^, Figure 1d. Otherwise, it is similar to the phenanthrene spectrum and shows the *n* = 4 magic peaks for [(An)_*m*_(H_2_O)_*n*_]^−^.

To demonstrate the magic numbers more clearly, we plot the integrated mass peak intensities of the hydrated clusters [(PAH)_*m*_(H_2_O)_*n*_]^−^ (*n* ≥ 1) in Figure 2. The ion abundances are represented by the integrated intensities rather than by the maximum peak intensities since the peak widths depend on *m*/*z*. We also omit the pure (PAH)_*m*_^–^ clusters from Figure 2 since they dominate all spectra except for the naphthalene spectrum. For Np and CNNp, the number of PAH molecules in Figure 2a,b corresponds to *m* = 1–4, while *m* = 2–5 for Ph and An in Figure 2c,d. The 3D plots clearly show the magic peaks *n* = 4 in all spectra, and also weaker *n* = 8 in some cases.

**Figure 2:**
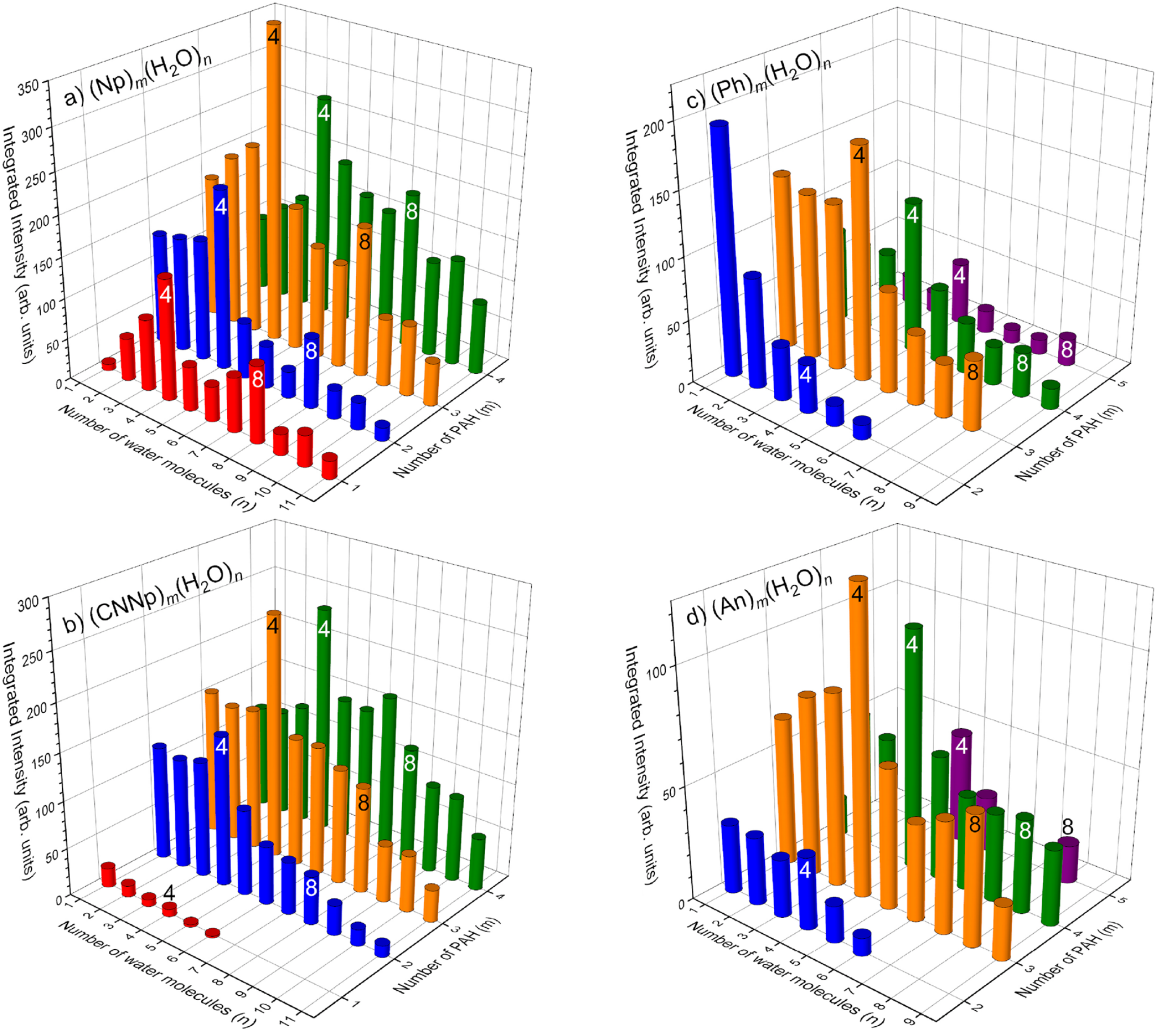
Integrated intensities of the [(PAH)_*m*_(H_2_O)_*n*_]^−^ clusters ion mass peaks from Figure 1 for Np (a), CNNp (b), Ph (c), and An (d). Magic peaks *n* = 4 and 8 are labeled.

### Calculations

We have explored the structures and stability of neutral Np-water clusters by DFT calculations. Although (Np)_1_(H_2_O)_*n*_ cluster structures have been reported in the literature for *n* ≤ 4 and *n* = 6,^43,44,46,52^ structures with more water units or those containing two Np molecules have not yet been discussed to the best of our knowledge. Figure 3 shows our calculated most stable structures of (Np)_*m*_(H_2_O)_*n*_, *n* = 1–9, (a) *m* = 1 and (b) *m* = 2. The structures of small clusters are consistent with previous reports.^43,44,46,52^ A single water molecule interacts via an OH···π bond with the aromatic ring rather than accepting the C–H bond by the O atom. Two water molecules can adopt several arrangements, shown in SI (Figure S6). Water molecules for *n* = 3–5 form cyclic hydrogen bonded structures with OH bonds that point toward naphthalene.^43,44,52^ For *n* = 6, a 3D water network is formed on top of naphthalene.^46^ A similar pattern appears for larger PAHs.^47,68^ The hydrogen-bonded water cluster attached by two OH···π bonds to naphthalene is also a preferred interaction pattern for *n* = 7–9. Such large aqueous clusters can form additional C–H···O bonds. This picture is analogous to coronene.^47^ We have also explored more uniform hydration patterns of a single naphthalene molecule by placing different numbers of water molecules on each side; however, all yielded higher energies (see SI, Figures S6–S8).

**Figure 3:**
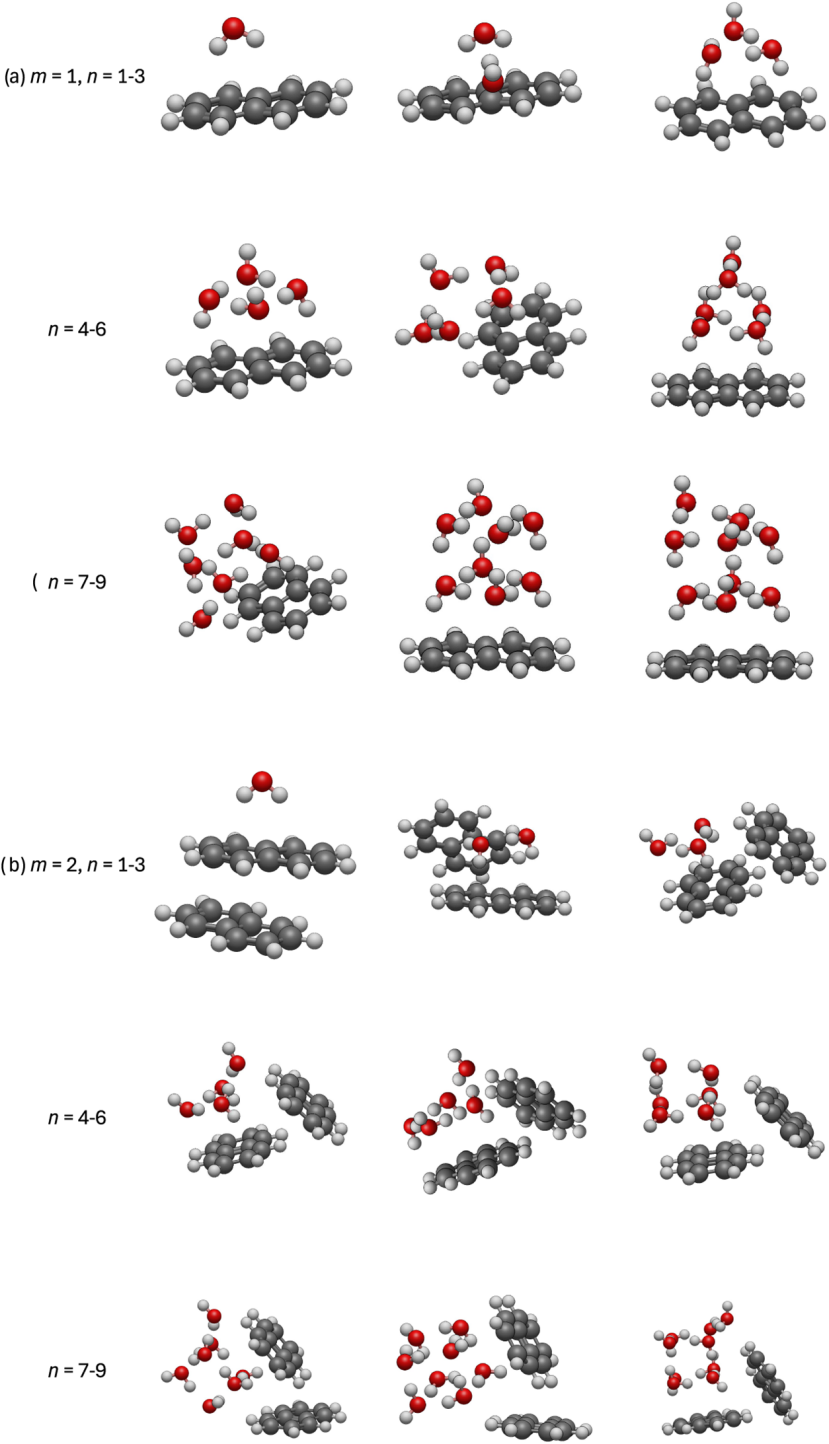
Most stable structures of (Np)_*m*_(H_2_O)_*n*_ for (a) *m* = 1 and (b) *m* = 2, with *n* = 1–9 water molecules. The structures for *m* = 1, *n* ≤ 4 and *n* = 6 agree well with the previously published ones.^43,44,46,52^ For more isomers see SI, Figures S6–S14.

All calculated lowest energy structures of (Np)_2_(H_2_O)_1–9_, Figure 3 (structures in lower three panels, b), possess an essentially unchanged water cluster of a given *n* interacting with the naphthalene dimer in various ways: with a single aromatic plane of the Np dimer or intervening between the tilted planes. Such patterns have also been observed for (pyrene dimer)-water clusters.^68^ Figures S9–S14 in SI indicate that the energy difference between these two motifs is not dramatically high, it does not exceed 0.17 eV and is lower for most *n*. Figures S9–S14 also show that for *n* ≥ 3, the water clusters can be attached from the side of the Np dimer and for *n* ≥4, the sandwich structure is also possible. The former pattern is similar to the pyrene analog in ref 68, and the sandwich structure resembles the confinement of water between larger PAHs investigated in refs 53,54 .

## Discussion

Two principal factors drive the abundances in the mass spectrum: (i) size distribution of the neutral clusters and (ii) how does this distribution change upon ionization. The size distribution of the neutral clusters results from the formation of cluster in supersonic expansion followed by cluster evaporation in vacuum. Due to the low hydration conditions, the amount of PAH molecules exceeds H_2_O in our expansion. Thus, collisions between PAH molecules and the stabilizing collisions with buffer gas Ar are more frequent than with water molecules. We propose that PAH clusters are generated first and mixed species are formed when the PAH cluster collides with the H_2_O molecule and water attaches to the cluster (eventually a PAH molecule can be evaporated). Thus, the mixed clusters with water are formed by sequential uptake of water by PAH clusters. The collisions forming the clusters in the expansion stop a few millimeters downstream from the nozzle throat, and then the clusters fly through vacuum without further collisions on a flight path of ≈1.5 m (≈2.5 ms). Cluster evaporation is governed by an evaporative ensemble that is determined by the binding energies of the cluster constituents.^71^

The electron attachment leads to deposition of an additional energy into the neutral clusters (kinetic energy of the electron plus electron affinity), which can be released by the evaporation of the cluster constituents. We argue above in the Experimental Section that mainly electrons with near-zero kinetic energies contribute to the electron attachment here. In addition, a relatively large amount of energy can be distributed over the many degrees of freedom of the clusters without leading to any cluster constituent evaporation. The anion abundance is then determined by the electron affinity, the binding energies of the constituents in the anionic cluster and the energy redistribution. We argue in the following that the present “magic numbers” point to higher stability of the corresponding *neutral* clusters of PAH with 4 and 8 water molecules.

The first reason is that a mass spectrum of [(Np)_1_(H_2_O)_*n*_]^−^ of Bower and co-workers^15^ does not show any evidence of the *n* = 4 magic number. Their spectrum (Figure 1 in ref 15) is shown only up to *n* = 4, but the intensity of [(Np)_1_(H_2_O)_*n*_]^−^ decreases monotonically from *n* = 2 to 4. The anions in ref 15 were generated in a microplasma in a supersonic expansion high-density region, where the ions undergo frequent collisions. On the other hand, in our experiment, the neutral clusters generated in supersonic expansion fly isolated in vacuum without collisions before they are ionized in a single collision with a slow electron. We have recently compared different anion generation in these two experiments for the case of naphthalene cluster anions and observed different abundances.^21^ When Np anion clusters were formed in microplasma,^17^ the dimer anion (Np)_2_^–^ was stabilized by collisions and observed. However, in our experiment,^21^ the (Np)_2_^–^ anion could not be generated in a single electron collision with the (Np)_2_ dimer or with the (Np)_3_ trimer. It was formed from mixed clusters (Ar)_*m*_(Np)_2_ with Ar, where the dimer anion (Np)_2_^–^ was stabilized by Ar evaporation after electron attachment. It is reasonable to assume that under microplasma conditions, where the anions undergo multiple collisions, the energetics of the anions is the main factor driving their abundances and it clearly does not lead to a higher stability of *n* = 4.

The second reason is the intensity pattern in the *positive* ion mass spectra of PAH-water clusters observed also in other experiments. There is clear evidence for a more pronounced higher abundance of the peak *n* = 4 in different mass spectra of positive ions [(PAH)_*m*_(H_2_O)_*n*_]^+^ in the literature. Xu et al.^52^ ionized naphthalene-water clusters with tunable vacuum ultraviolet (VUV) photoionization using synchrotron radiation. Their spectra (Figure 1 Figure 1 in ref 52) exhibit a somewhat higher intensity of [(Np)_*m*_(H_2_O)_*n*_]^+^ ions for *n* = 4. Lemmens et al.^43^ ionized the naphthalene-water clusters using the REMPI technique with excitation at 32,432 cm^–1^ and ionization at 193 nm. Their mass spectrum (Figure S1 in their Supporting Information) shows cluster ions [(Np)_*m*_(H_2_O)_*n*_]^+^ with a strong propensity for the water tetramer *n* = 4 with *m* = 1–3 Np molecules. Evidence for a mass peak of higher abundance with *n* = 4 water molecules can be found in the VUV-ionized (photon energy of 10 eV) anthracene-water clusters [(An)_*m*_(H_2_O)_*n*_]^+^ (Figure 1 in ref 53). Thus, a higher abundance of mixed PAH-water clusters with four water molecules was observed in the positive ion mass spectra for different clusters, formed under different conditions and ionized by different methods; however, none of these references 43,52,53 discussed the higher abundance of the peak *n* = 4.

Magic clusters with four water molecules were mentioned in the study of ion–molecule reactions in He nanodroplets doped with fullerens and heavy water, where local abundance maxima appeared for [(C_60_)_*x*_(D_2_O)_4_]^+^, *x* = 1 and 2.^72,73^ This was explained by an enhanced evaporation energy of the neutral water tetramer. The cationic complexes resembled water clusters weakly bound to C_60_^+^. Theoretical study of water clusters on cationic carbonaceous seeds confirmed a higher relative stability of [(PAH)­(H_2_O)_4_]^+^.^74^ The behavior of relative stabilities followed that of the corresponding pure water clusters and was quite similar to the one found for models of neutral water-C_60_.^75^ Investigation of the uptake and accommodation of water clusters by adamantane clusters in helium droplets revealed the interplay of magic number water clusters with magic number adamantane clusters^76^ and local maxima with 4 water molecules were again observed.

We have also measured the mass spectra in the positive ion mode of our RTOF ionizing the clusters with 70 eV electrons. Although the positive ion spectra (shown in SI, Figures S1–S4) are congested by substantial cluster fragmentation, the abundance of [(PAH)_*m*_(H_2_O)_*n*_]^+^, *n* = 4, is slightly higher than that of neighboring mass peaks *n* = 3,5 for Np, An, and Ph in our spectra as well.

Thus, a higher abundance of [(PAH)_*m*_(H_2_O)_*n*_]^+^ cluster ions with *n* = 4 water molecules was observed in different studies of positive ions. Here, we show that the *n* = 4 magic number is observed also in the negative ion spectra. This suggests that it originates from the higher abundance and thus stability of the corresponding neutral clusters with the water tetramer. The currently observed (weaker) magic number *n* = 8 opens the question of whether it corresponds to clusters with two separate water tetramers or whether there is a higher stability of the water octamer attached to PAH clusters. This question was also addressed by our theoretical calculations.

To characterize the stability of the clusters with respect to the loss of a water molecule, we focus on the incremental binding strengths: (Np)_*m*_(H_2_O)_*n*_ → (Np)_*m*_(H_2_O)_*n*−1_ + H_2_O. We have chosen such a quantity because the differences for specific *n* are clearer than in the case of the absolute binding strength that yields a monotonous curve with small bumps (Figure S16 in SI). The energy differences (including the zero-point vibrational energy) at the ωB97XD/aug-cc-pvdz are shown in Figure 4. Note that the effect of the size of the basis set is not significant, Figure S17 shows that employing aug-cc-pvtz decreases the incremental binding strength by less than 0.03 eV. The solid symbols and lines correspond to the minimum energy structures shown in Figure 3. The binding strength of the first water molecule to a single Np at the level ωB97XD/aug-cc-pvdz lies between the values reported in ref 52 and ref 44.

**Figure 4:**
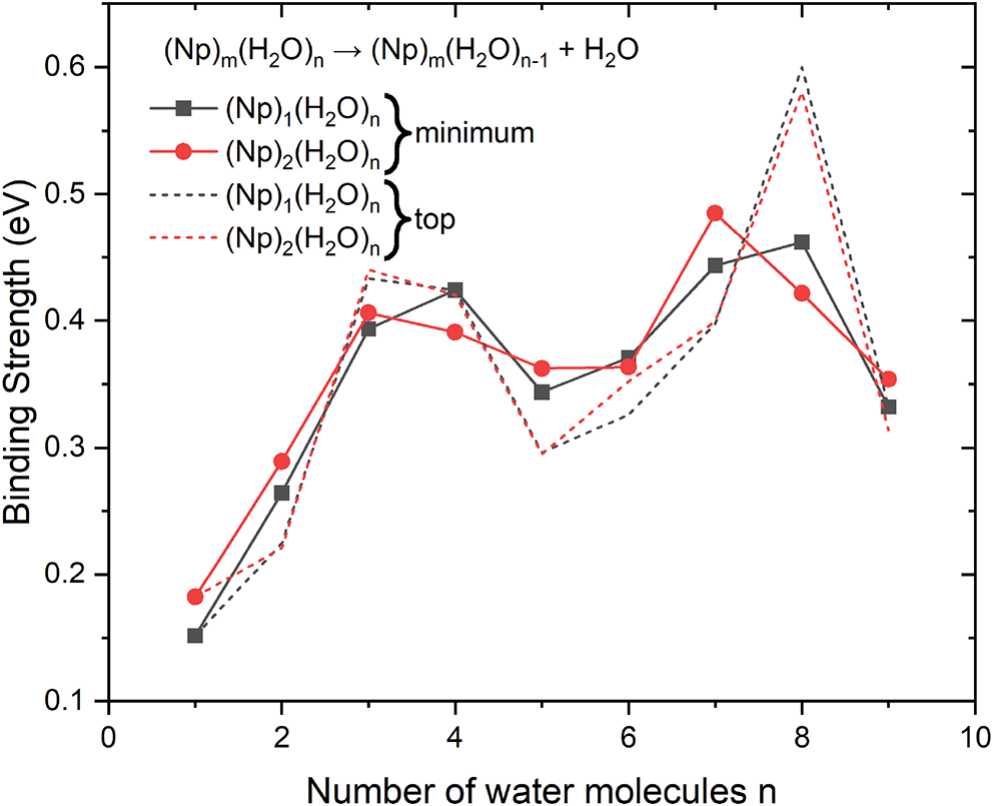
Binding strength of (Np)_*m*_(H_2_O)_*n*_ clusters for *m* = 1 and 2 and *n* = 1–9. The binding strength corresponds to the process outlined at the top. The values are connected by line for guiding the eye. Solid lines and symbols correspond to the minimum energy structures (Figure 3), dashed lines to the “on top” structures (see SI).

Water binding strength increases with the number of water molecules to more than 0.4 eV for *n* = 4, then decreases to a local minimum at *n* = 5. The second maximum is located at *n* = 8 and corresponds to 0.46 eV. The water binding strengths for the dimer (Np)_2_(H_2_O)_*n*_ also possess two maxima similarly to the monomer; but they occur for *n* = 3 and for *n* = 7. Their binding strengths are 0.41 eV (just 0.02 eV larger than that of *n* = 4) and 0.48 eV (0.06 eV larger than that of *n* = 8). This is presumably caused by the favorable stabilizing interactions for the odd-number water clusters intervening between the two tiled Np planes. In addition to two hydrogen bonds OH···π toward one Np, there are two C–H bonds pointing toward the water oxygen atoms. These clusters are further stabilized by the C–H bonds of one Np pointing toward the aromatic rings of the second Np. The presence of all these attractive interactions in turn modulates the Np–Np arrangement.

In supersonic expansions, clusters are formed by adding water molecules in subsequent collisions. The colliding water molecule will extend the water network bound by the strong hydrogen bonds rather than annealing the cluster to its minimum structure. Thus, we also evaluated binding strengths for the structures where an essentially unperturbed hydrogen bonded water cluster is placed on top of the aromatic rings of Np to which it is bound via the OH···π bonds (see highlighted structures in SI). It is the most stable structure for clusters that contain a smaller number of water molecules. In other cases, it lies at most 0.17 eV above the minimum (see SI). These structures exhibit a strong maximum for *n* = 8 determined by the stability of the water octamer, see dashed lines in Figure 4. For reference, we have also calculated the incremental and absolute binding strengths of the pure water clusters (see SI, Figure S18). Generally, multiple cluster isomers are generated in supersonic expansions, and our calculations suggest several energetically accessible isomers for the present clusters (Np)_*m*_(H_2_O)_*n*_. The energetically low lying structures exhibit on average a higher stability for *n* = 4 and 8.

The higher stability of PAH complexes with *n* = 4, 8 water molecules suggests their higher abundance in the molecular beam. Upon electron attachment, the electron is localized on the Np moiety.^15,32^ Such charge distribution induces structural changes in the attached water cluster. The position of oxygen atoms is roughly preserved, but the direction of the hydrogen bonds changes, so that number of O–H bonds pointing toward the PAH is maximized. This has been already reported for [(Np)_1_(H_2_O)_1–6_]^−^.^32^ We have performed calculations for [(Np)_1_(H_2_O)_7–9_]^−^ (Figure S19 in the SI) that confirm this way of hydrogen bond rearrangement. As anticipated, the sum of Mulliken charges of Np atoms is about −1.

The nominal energy of our electron beam is about 2 eV, however, it also contains near-zero electrons, as discussed in the Experimental section. These low energy electrons are likely to be attached to the clusters since both PAHs and water exhibit zero energy resonances.^8,14^ Thus, the energy released into the cluster after the electron attachment mainly corresponds to the electron affinity. The measured adiabatic electron affinity of (Np)_1_(H_2_O)_*n*_ increases smoothly with the hydration level from 0.11 eV for *n* = 1 to 1.5 eV for *n* = 8.^15^ Apparently, this excess energy given to the system in attachment processes modifies the size distribution only slightly, so that the high abundance of *n* = 4 and 8 is conserved. According to the calculated binding strengths, *n* = 8 should be more pronounced than *n* = 4 in the neutral clusters. However, two effects suppress the abundance of the larger clusters. First, in the expansion regime used in the present study, small clusters are preferred, and the abundance of larger clusters quickly decreases because of the insufficient number of collisions in the weak expansion. Thus, any dependence of cluster stability on size must be modified by an exponential decrease in cluster size *n*. Second, the electron affinity of about 1.5 eV for *n* = 8 is almost twice as large as for *n* = 4 (see Figure 3 in ref 15). Thus, the evaporation induced by electron attachment for the larger sizes around *n* = 8 will be stronger and eliminate its dominance.

## Conclusions

In conclusion, we present mass spectra of the negative ions of the [(PAH)_*m*_(H_2_O)_*n*_]^−^ clusters formed by near-thermal electron attachment to the mixed PAH-water clusters produced in supersonic expansions. The spectra show pronounced magic numbers *n* = 4 for almost all *m* and all PAHs (naphthalene, 1-cyanonaphthalene, phenanthrene, and anthracene). This is interesting, especially since the pure negatively charged water tetramer (H_2_O)_4_^–^ is difficult to generate.^10^ Another magic number *n* = 8 is observed and is pronounced especially for Np. We conclude that the reason for the high abundance of these cluster sizes is the higher stability of the neutral (PAH)_*m*_(H_2_O)_*n*_ clusters with *n* = 4 and 8 water molecules. This leads to a high abundance of these cluster sizes in the neutral beam. The size distribution is little influenced by the electron attachment. In general context, the present results provide insight into the onset of complex aggregation that occurs in extraterrestrial environments as part of ice-grain formation and in planet’s atmospheres, including Earth, where water condenses to aerosols on carbonaceous molecules and seed particles. It would be interesting to confirm the magic numbers for clusters of larger PAH molecules with water, which would resemble more closely graphene structures in future investigations.

## Supplementary Material



## Data Availability

All data are publicly available in national public repository at 10.48700/datst.fr720-vq953.

## Notes

Integrated peak intensities and additional mass spectra of negative and positive cluster ions, and calculated cluster structures and energies (PDF)
