# Supplementary material for: Cluster Anions of Hydrated Polycyclic Aromatic Hydrocarbons: “Magic” Water Tetramer
Source: J Phys Chem A. 2026 Mar 30;130(14):2883–92. doi: 10.1021/acs.jpca.6c00211 (PMC13071915; doi:10.1021/acs.jpca.6c00211)
Supplement: Supplementary file 1 [file jp6c00211_si_001.pdf]

Cluster Anions of Hydrated Polycyclic Aromatic Hydrocarbons: “Magic” Water Tetramer

Jozef Ďurana,[†,‡] Barbora Kocábková,[†,‡] Andrij Pysanenko,[†] Eva Pluhařová,[†] Juraj Fedor,[†] and Michal Fárník[*,†]

†*J. Heyrovský Institute of Physical Chemistry, v.v.i., Czech Academy of Sciences, Dolejškova 2155/3, 182 23 Prague, Czech Republic*

‡*Also affiliated at: University of Chemistry and Technology Prague, Technická 5, 166 28 Prague 6, Czech Republic*

E-mail: michal.farnik@jh-inst.cas.cz

Integrated peak intensities

Table S1: Integrated peak intensities (arbitrary units)

Peak series identity	Number of water molecules (n)										
	1	*2*	*3*	*4*	*5*	*6*	*7*	*8*	*9*	*10*	*11*
$(Np)(H_2O)_n^-$	8	52	88	148	54	43	67	93	25	38	22
$(Np)_2(H_2O)_n^-$	135	141	148	220	69	52	33	86	35	33	16
$(Np)_3(H_2O)_n^-$	175	210	233	381	176	137	127	182	82	86	53
$(Np)_4(H_2O)_n^-$	93	117	138	272	201	170	159	191	117	130	87
$(CNNp)(H_2O)_n^-$	20	12	7	8	3	2	–	–	–	–	–
$(CNNp)_2(H_2O)_n^-$	120	116	122	158	90	61	55	51	31	17	12
$(CNNp)_3(H_2O)_n^-$	153	145	149	255	136	136	121	111	60	60	34
$(CNNp)_4(H_2O)_n^-$	110	113	127	239	150	148	169	123	92	89	55
$(Ph)_2(H_2O)_n^-$	193	88	43	39	16	11	10	9	–	–	–
$(Ph)_3(H_2O)_n^-$	138	131	131	182	80	55	42	55	–	–	–
$(Ph)_4(H_2O)_n^-$	73	68	71	120	59	41	31	35	17	–	–
$(Ph)_5(H_2O)_n^-$	9	22	16	49	18	11	12	21	–	–	–
$(An)_2(H_2O)_n^-$	30	30	25	32	16	8	–	11	–	–	–
$(An)_3(H_2O)_n^-$	65	79	84	132	61	43	49	27	23	–	–
$(An)_4(H_2O)_n^-$	16	59	53	105	54	42	39	41	33	–	–
$(An)_5(H_2O)_n^-$	–	–	–	48	24	–	–	17	–	–	–

Positive ion mass spectra

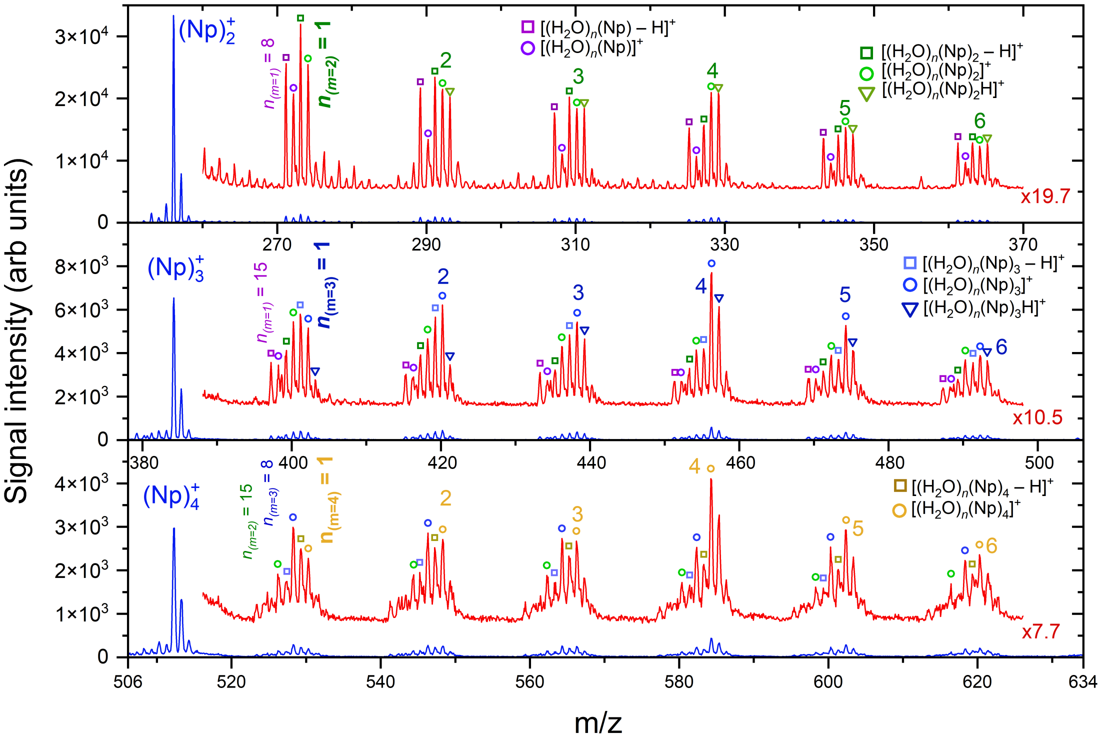

Fig. S1: Positive ion mass spectrum of hydrated Np clusters at E_e = 70 eV. The spectra are shown from the mass range of naphthalene dimer since the region below m/z ≈ 200 is obscured by the TOF background noise at lower signal intensities. The spectra consist of the main $(Np)_m^+$ series and $(Np)_m(H_2O)_n^+$ series of lesser intensities (these are shown in greater detail in spectra colored red). The individual water series are comprised of "triplet" peaks, corresponding to pure $(Np)_m(H_2O)_n^+$, deprotonated $[(Np)_m(H_2O)_n{-}H]^+$ and protonated $[(Np)_m(H_2O)_n^+H]$ fragments of various intensities. Furthermore, these triplets, to an extent, mass coincide with other water series. Isotopic distributions of carbon atoms also complicate the assignment, but they alone do not explain the higher intensities of some peaks, especially in water series with small m. At higher m, the pure $(Np)_m(H_2O)_n^+$ fragments dominate and the peaks with mass +1 can be more or less attributed to the isotopic distributions.

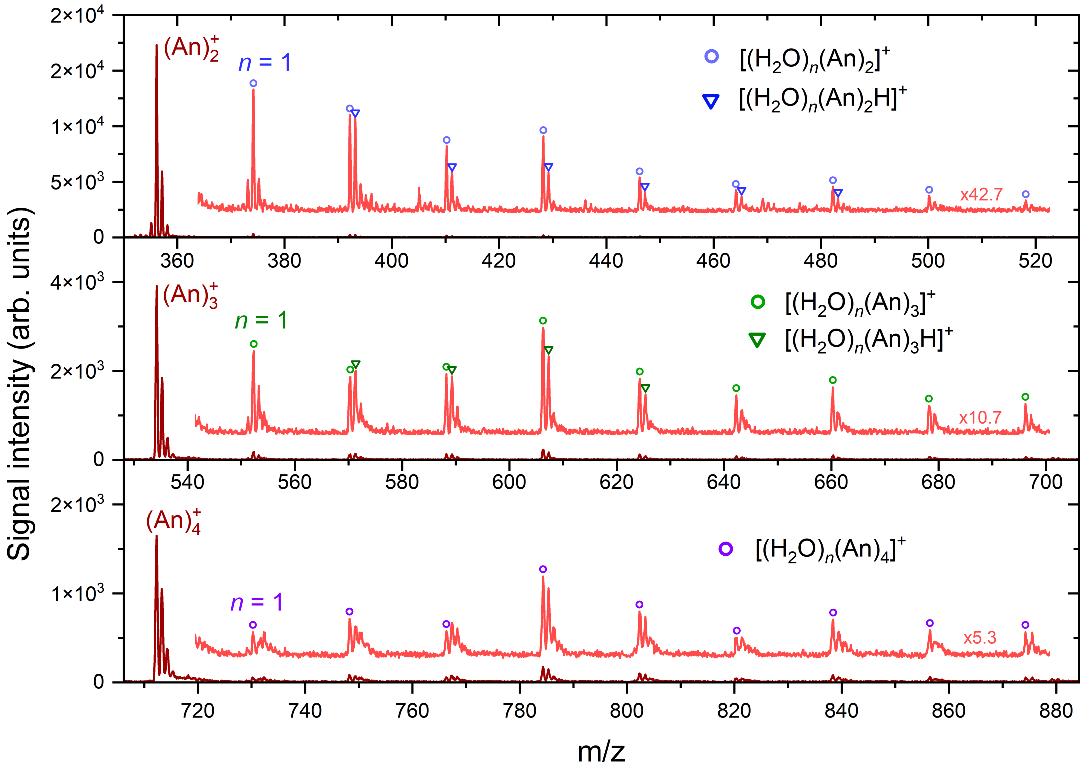

Fig. S2: Positive ion mass spectrum of hydrated An clusters at $E_e = 70$ eV. The spectra are shown from the mass range of the anthracene dimer, again, in order to avoid the peaks coming from the TOF background at the intensities of interest. Similarly to naphthalene, the spectrum consists of the pure $(An)_m^+$ series and lesser $(An)_m(H_2O)_n^+$ water series. In contrast to naphthalene, however, almost no deprotonated peaks are present (basically only visible with the pure anthracene series and with water series at $n = 1$). Protonated fragments are present only in a few instances, e.g., $(An)_2(H_2O)_{2–7}^+$ and $(An)_3(H_2O)_{2–5}^+$. Considering error intervals, all the other +1 mass peaks can be explained by isotopic distribution alone.

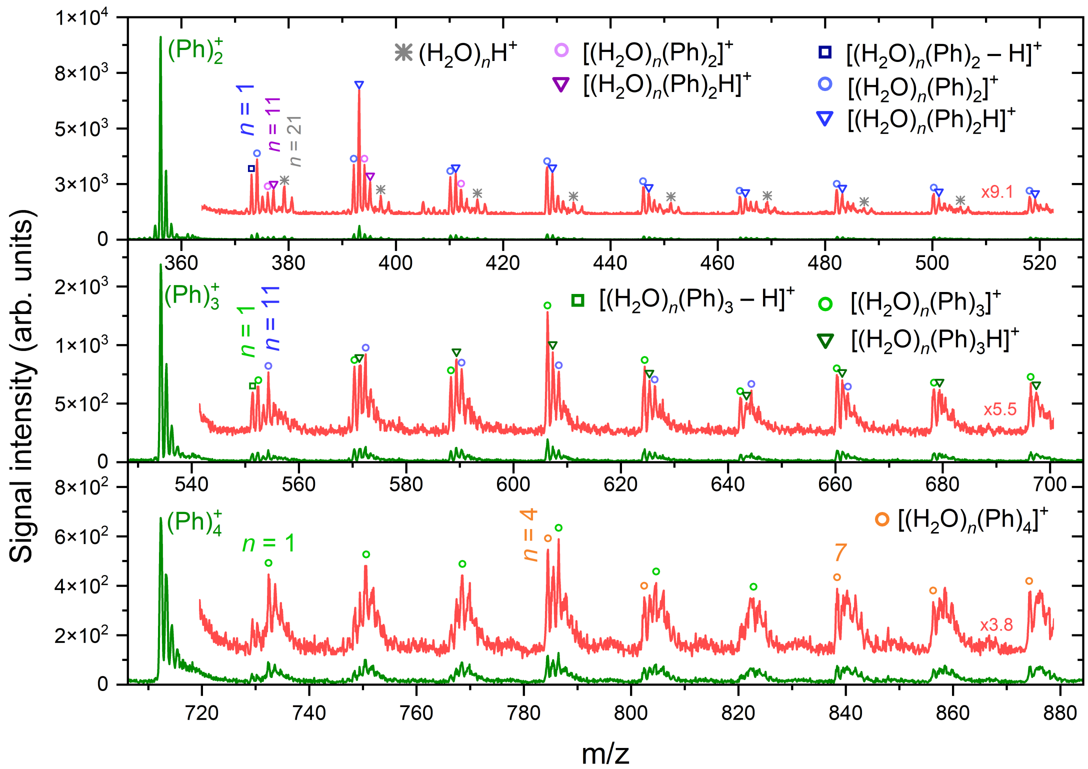

Fig. S3: Positive ion mass spectrum of hydrated Ph clusters at $E_e = 70$ eV. The spectra are also shown from the mass range of phenanthrene dimer. The spectra are relatively complex and similar to those of naphthalene. The combination of lower signal-to-noise ratio, isotopic distribution, overlap of individual series, and gradual appearance of metastable fragments make the unambiguous assignment of all peaks problematic, more so at higher masses. However, similar patterns to An and Np can be observed here as well, e.g., pronounced relative intensity of $(\mathrm{Ph})_m(\mathrm{H_2O})_4^+$ (especially for $m \geq 3$), abrupt appearance of protonated fragment $(\mathrm{Ph})_m(\mathrm{H_2O})_n\mathrm{H}^+$ at $n \geq 2$ (especially at lower m), etc.

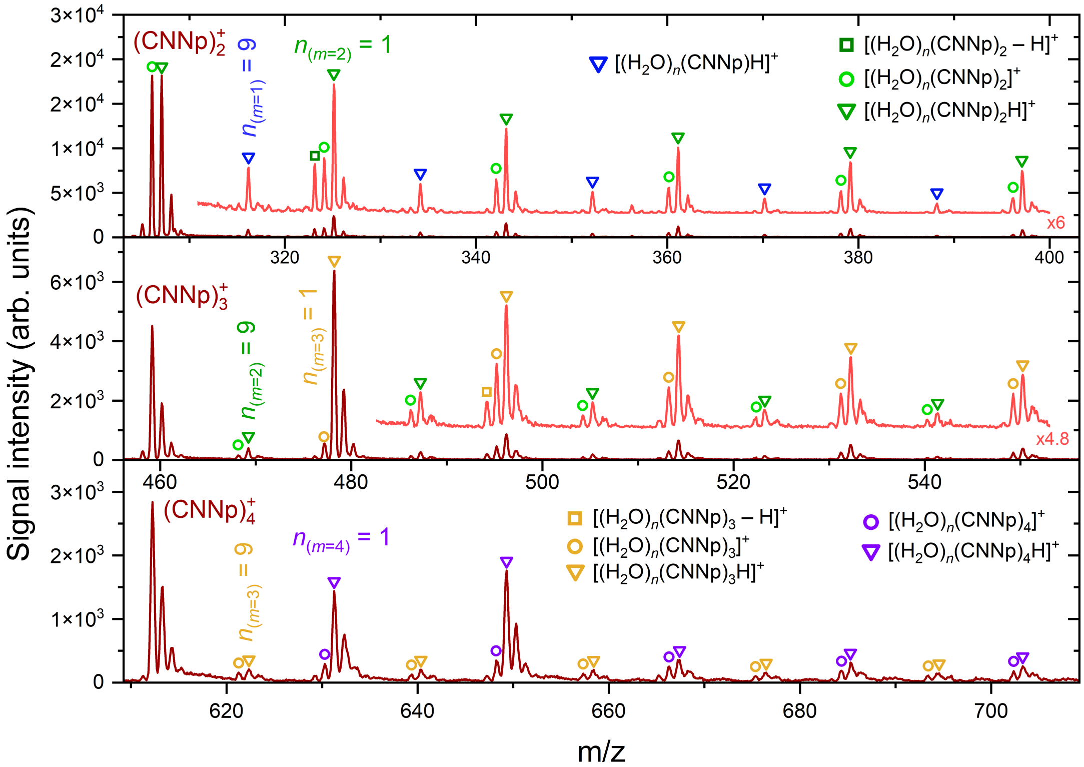

Fig. S4: Positive ion mass spectrum of hydrated CNNp clusters at $E_e = 70$ eV. The spectra are also shown from the mass range of the cyanonaphthalene dimer. Interestingly, cyanonaphthalene shares the same fragment series with Np, Ph, and An; however, no pronounced intensity at $(CNNp)_m(H_2O)_4^+$ is present.

Additional negative ion mass spectra

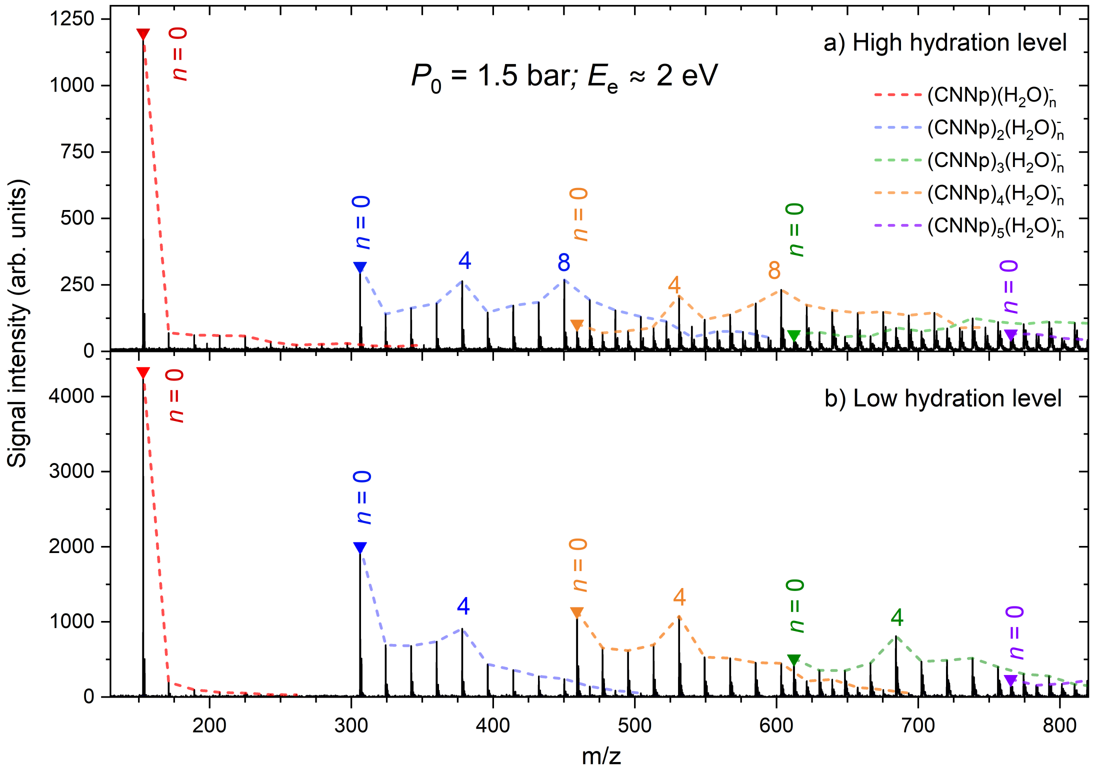

Fig. S5: Negative ion mass spectra of hydrated clusters of CNNp at $E_e \approx 2$ eV at a) high hydration level (Nafion membrane submerged in water) and b) low hydration level (Nafion membrane close above the water surface). Two differences can be immediately spotted: water series span much longer at high hydration level, to such a point that the series start to coincide (mass of $(H_2O)_{17}$ is equal to the mass of $(CNNp)_2$). Furthermore, high hydration levels also lead to pronounced intensity of $(CNNp)_m(H_2O)_8^-$ fragments.

Calculated structures of hydrated naphthalene monomer and dimer

The structures of hydrated naphthalene optimized at the ωB97XD/aug-cc-pvdz level of theory are shown in order of increasing energy. The energetically lowest isomer is always marked "_A" and its name is underlined in the following figures. The structures highlighted by the purple box are the most stable "on-top" isomers and were used to calculate the alternative incremental binding strength (dashed line in Fig. 4 in the article). All optimized geometries are available in xyz format in a public repository (https://doi.org/10.48700/datst.fr720-vq953) under the name given above each structure.

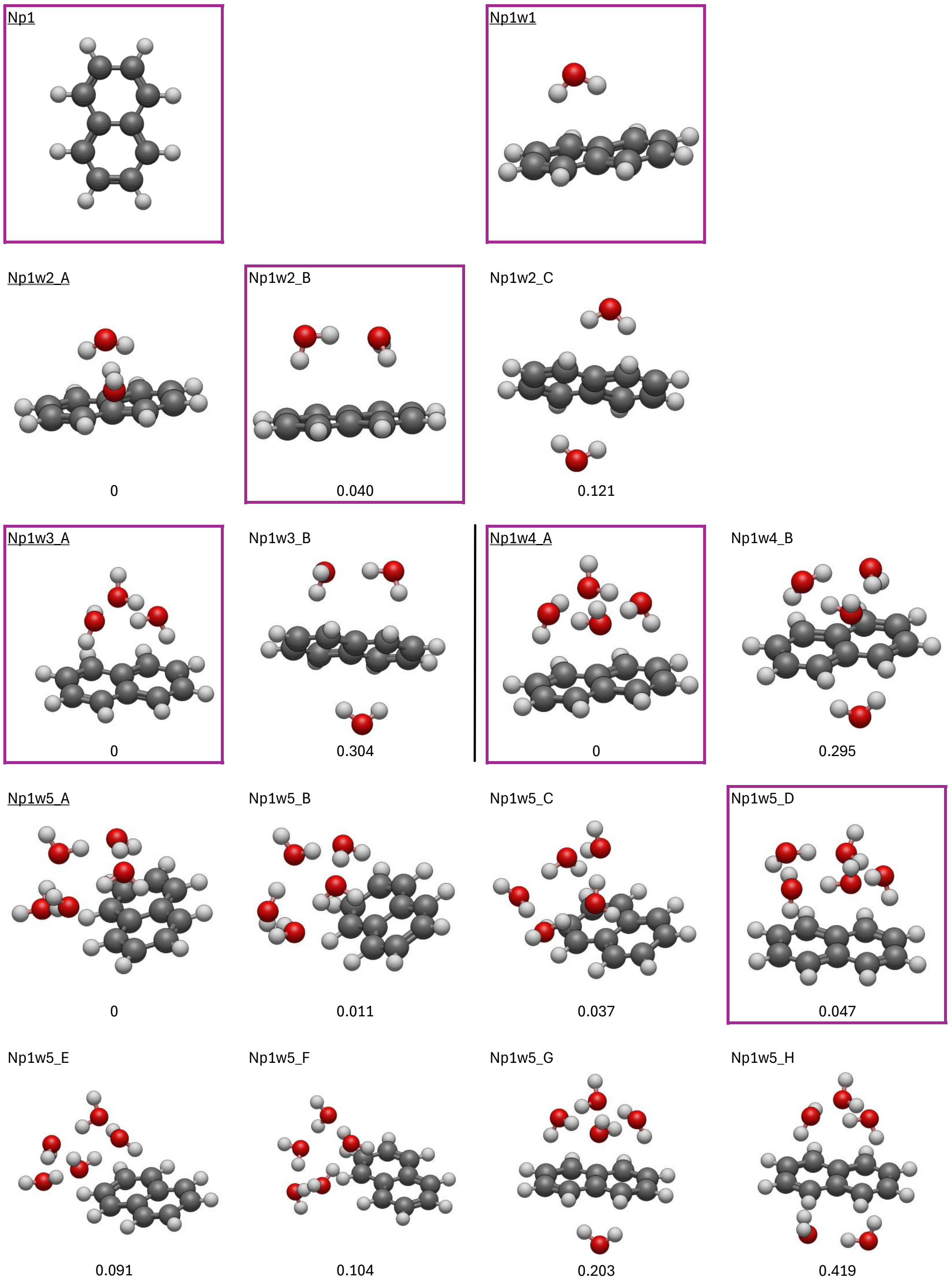

Fig. S6: Isomers of $(Np)_1(H_2O)_{1-5}$ optimized at ωB97XD/aug-cc-pvdz level of theory, energy given below each structure is in eV and includes the zero-point vibrational energy.

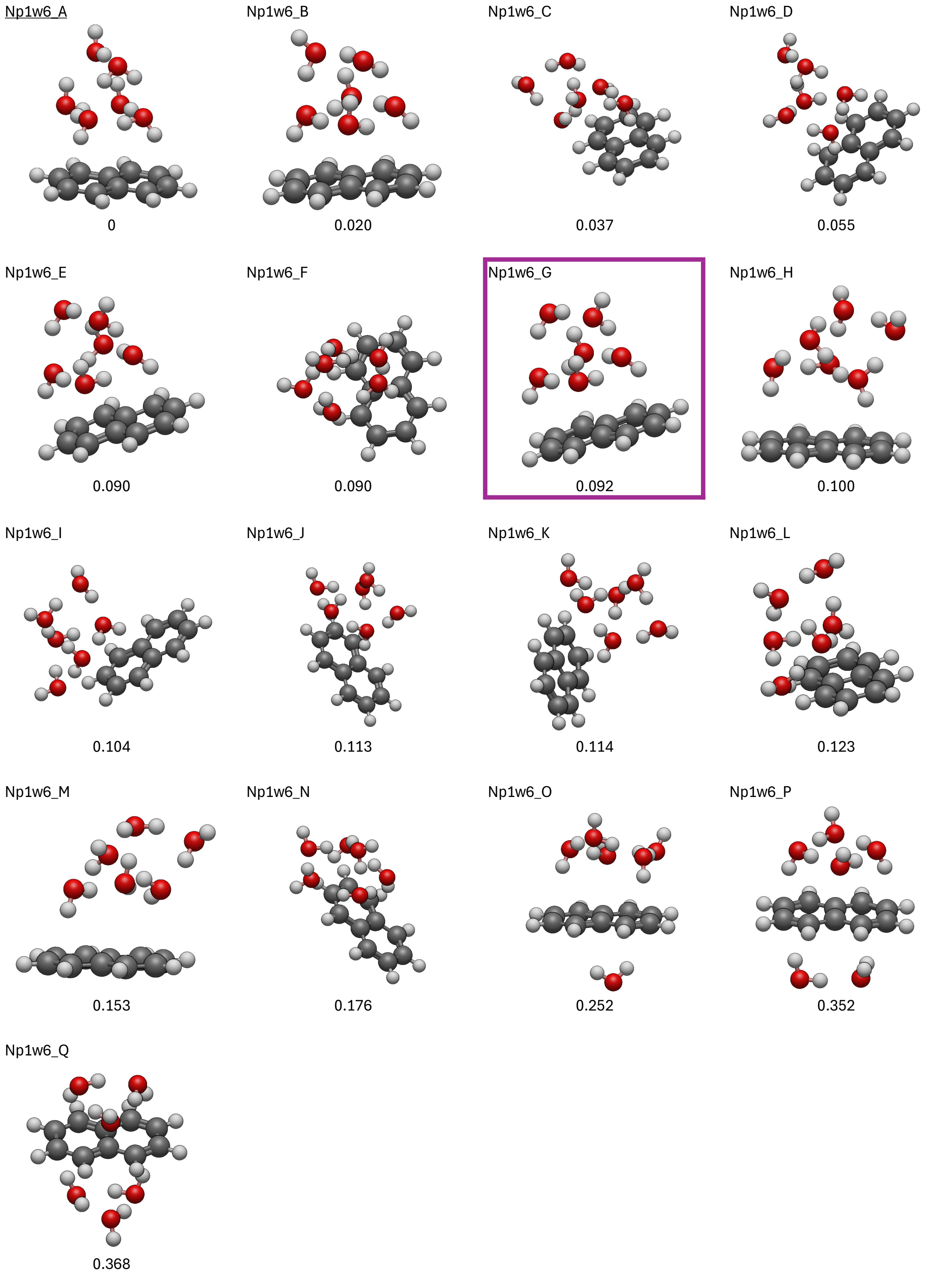

Fig. S7: Isomers of $(Np)_1(H_2O)_6$ optimized at ωB97XD/aug-cc-pvdz level of theory, energy given below each structure is in eV and includes the zero-point vibrational energy.

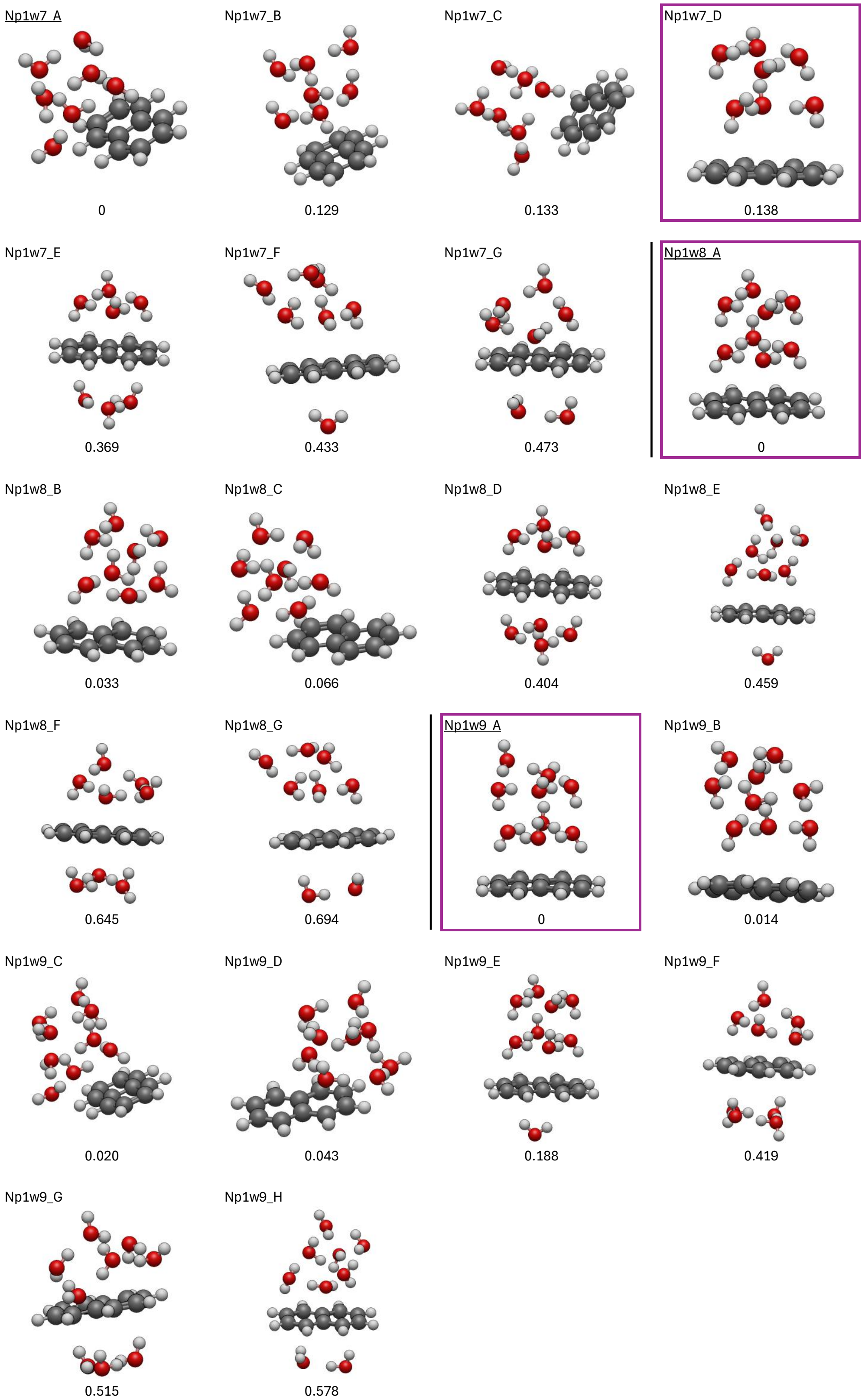

Fig. S8: Isomers of $(Np)_1(H_2O)_{7-9}$ optimized at ωB97XD/aug-cc-pvdz level of theory, energy given below each structure is in eV and includes the zero-point vibrational energy.

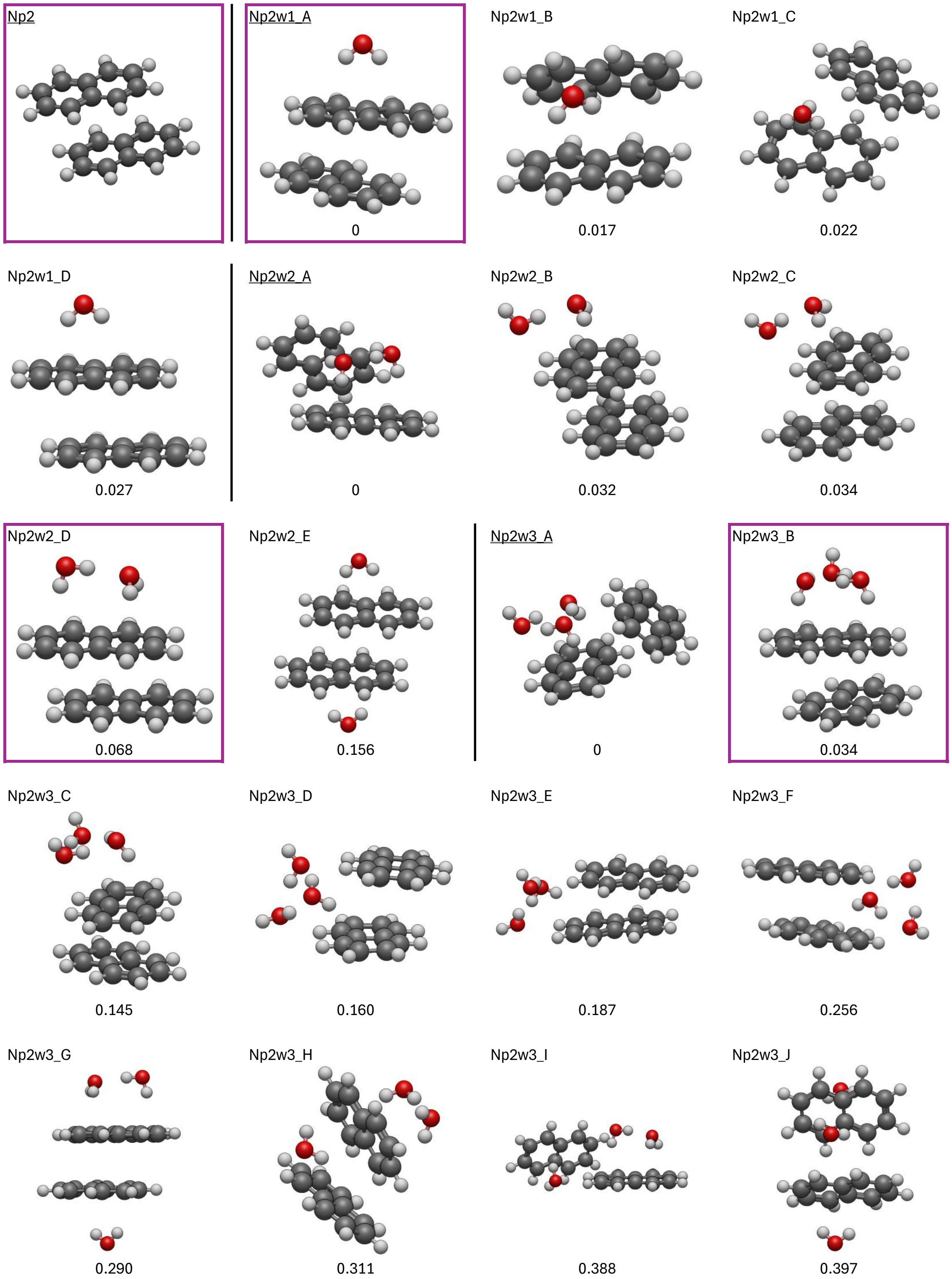

Fig. S9: Isomers of $(Np)_2(H_2O)_{1-3}$ optimized at ωB97XD/aug-cc-pvdz level of theory, energy given below each structure is in eV and includes the zero-point vibrational energy.

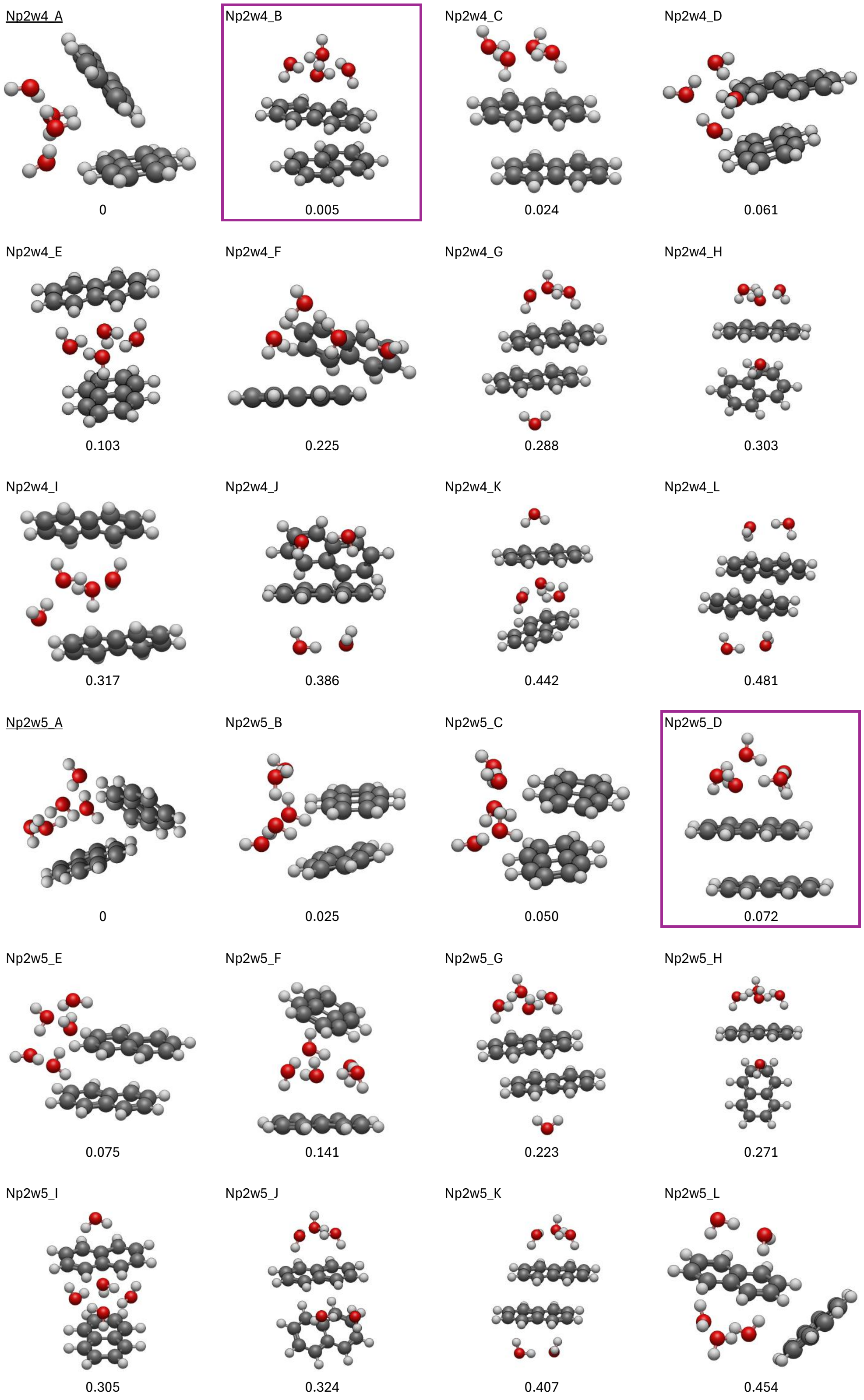

Fig. S10: Isomers of $(Np)_2(H_2O)_{4-5}$ optimized at ωB97XD/aug-cc-pvdz level of theory, energy given below each structure is in eV and includes the zero-point vibrational energy.

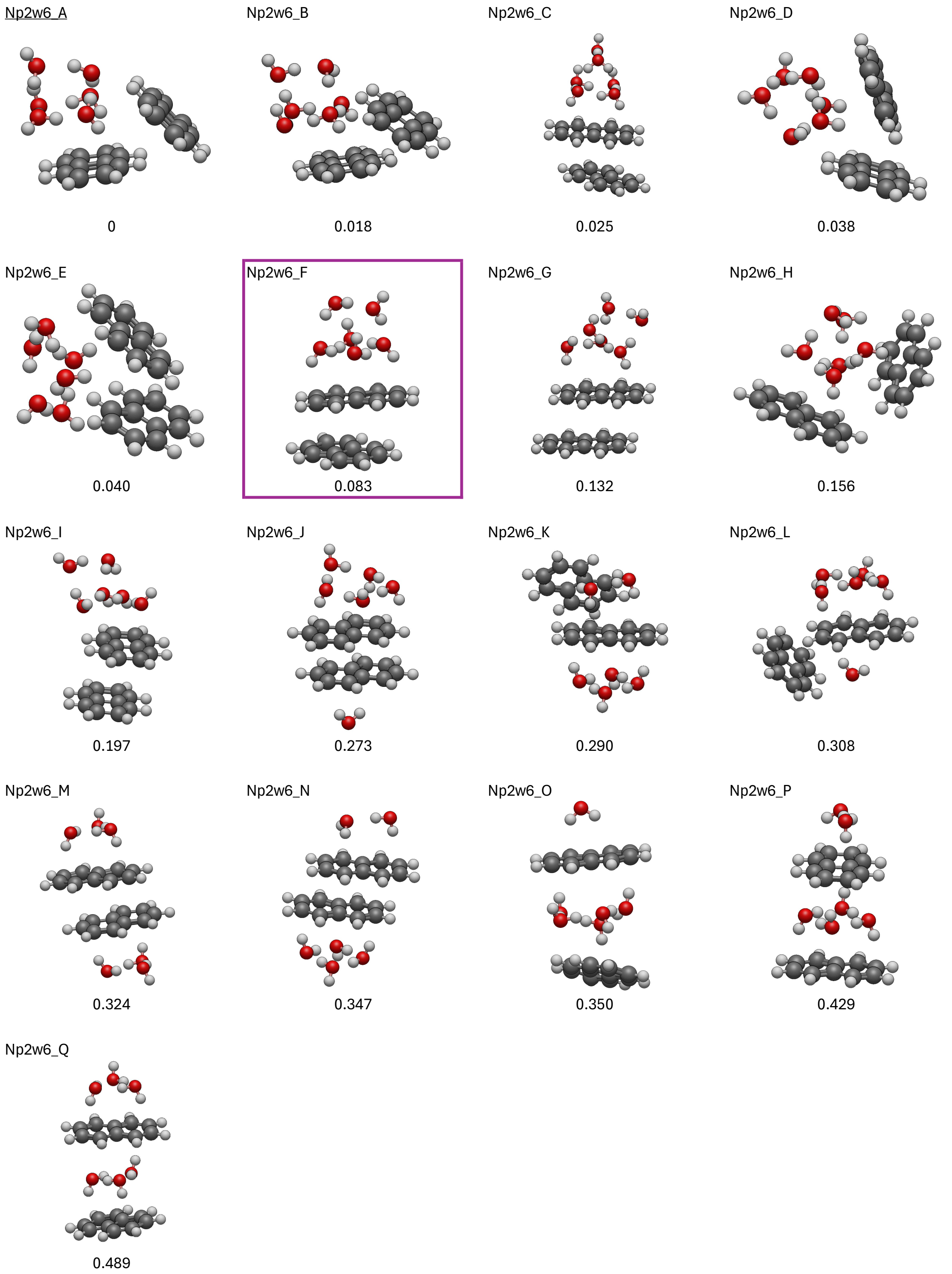

Fig. S11: Isomers of $(Np)_2(H_2O)_6$ optimized at ωB97XD/aug-cc-pvdz level of theory, energy given below each structure is in eV and includes the zero-point vibrational energy.

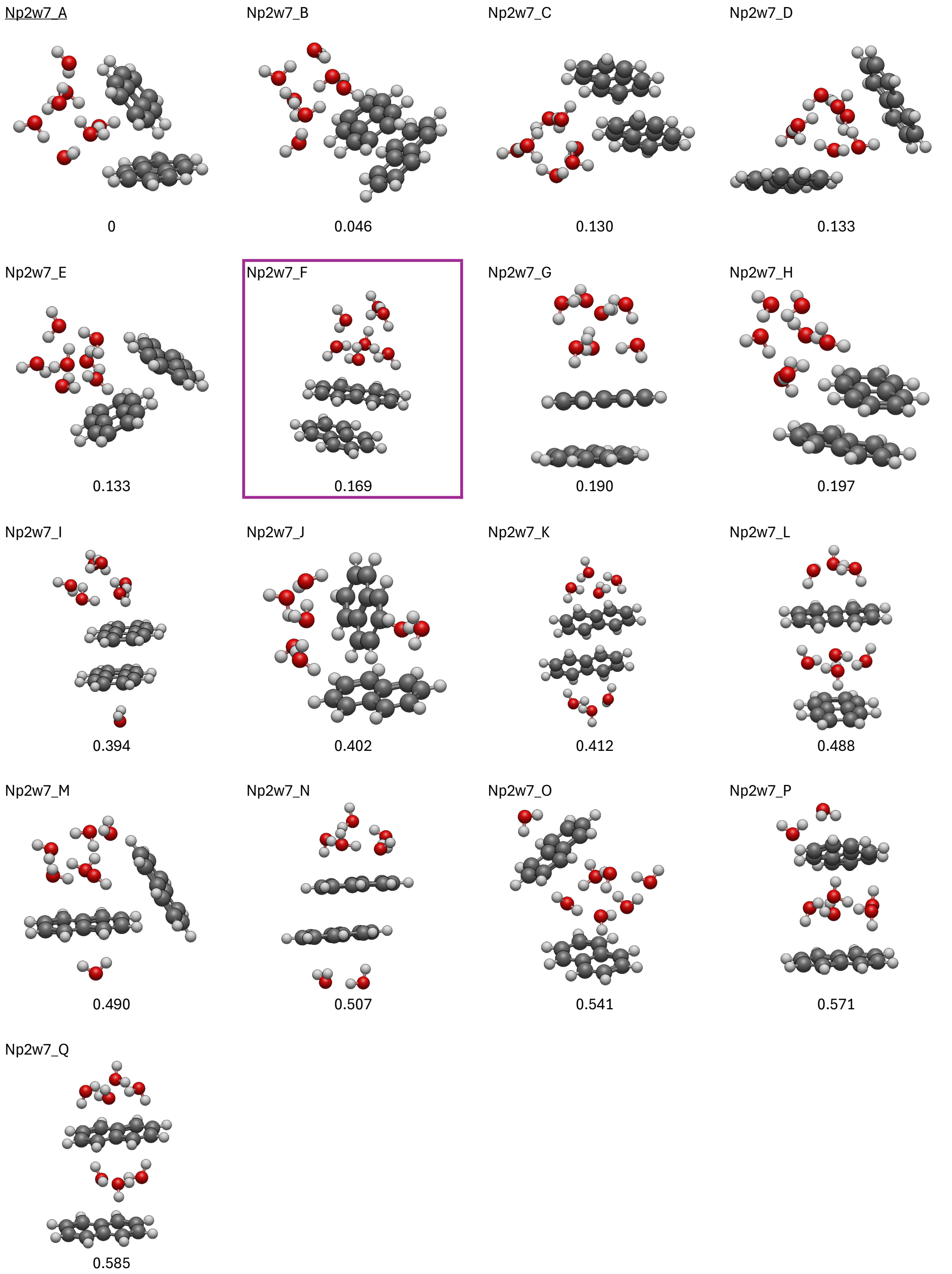

Fig. S12: Isomers of $(Np)_2(H_2O)_7$ optimized at ωB97XD/aug-cc-pvdz level of theory, energy given below each structure is in eV and includes the zero-point vibrational energy.

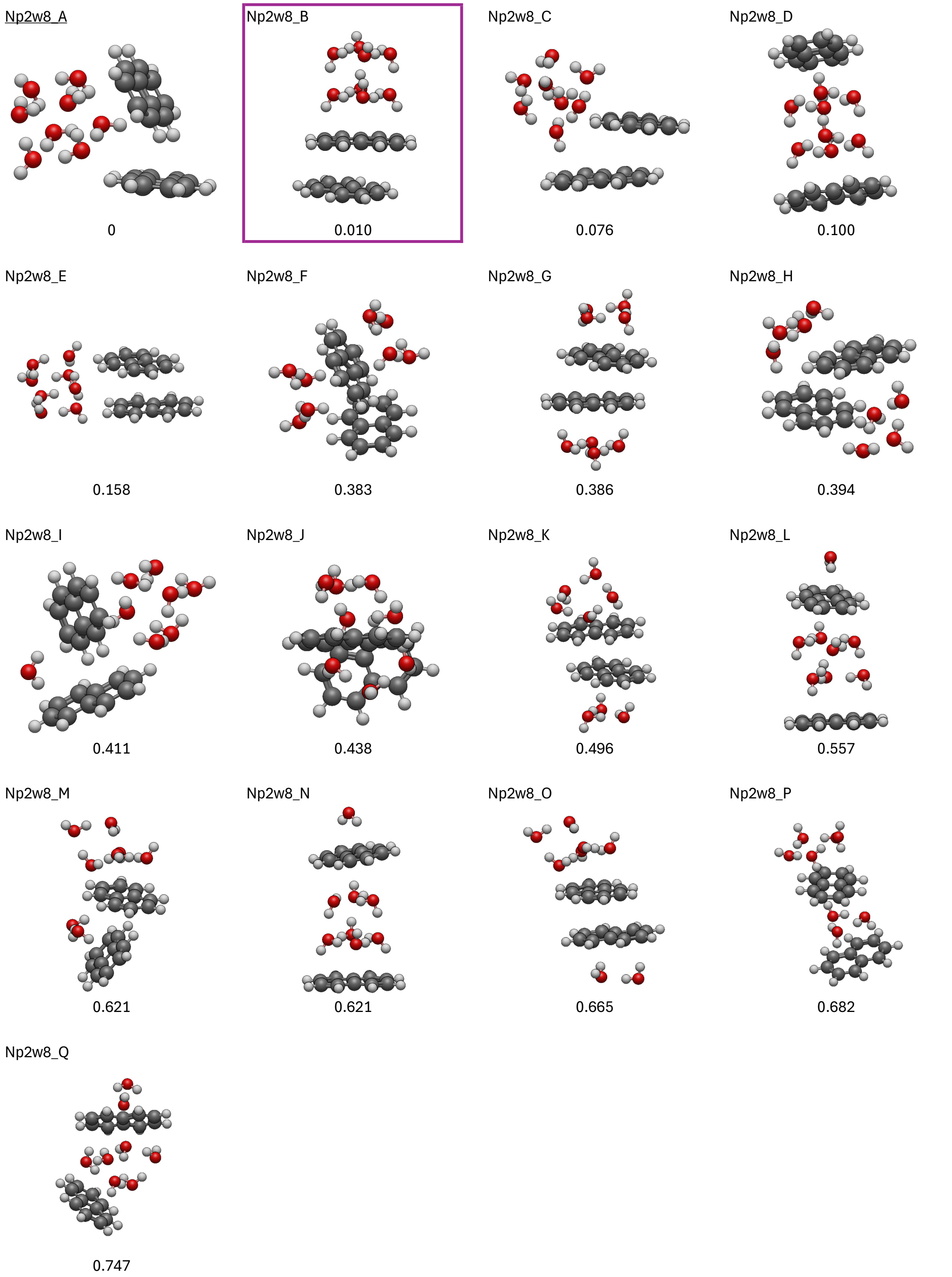

Fig. S13: Isomers of $(Np)_2(H_2O)_8$ optimized at ωB97XD/aug-cc-pvdz level of theory, energy given below each structure is in eV and includes the zero-point vibrational energy.

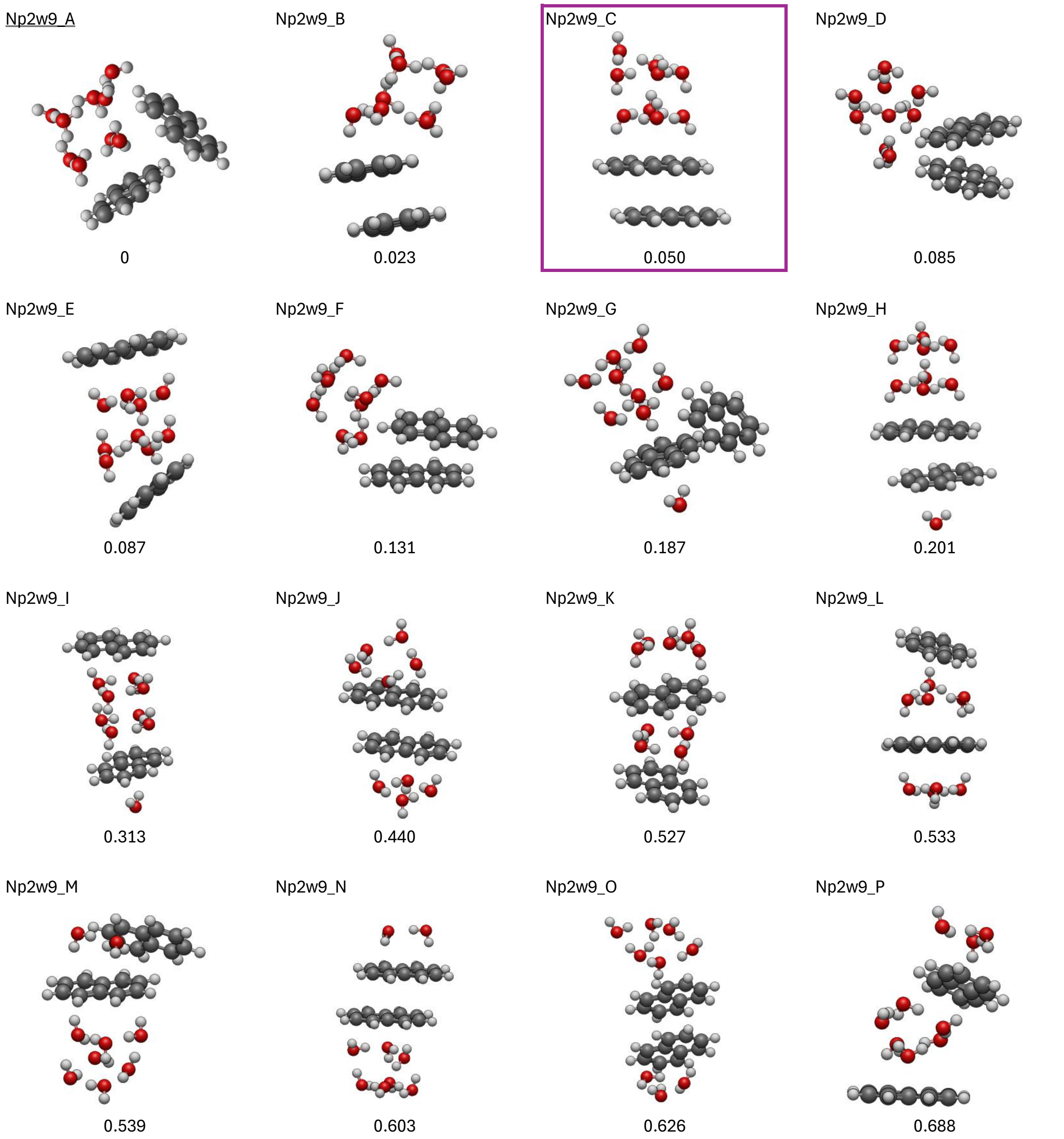

Fig. S14: Isomers of $(Np)_2(H_2O)_9$ optimized at ωB97XD/aug-cc-pvdz level of theory, energy given below each structure is in eV and includes the zero-point vibrational energy.

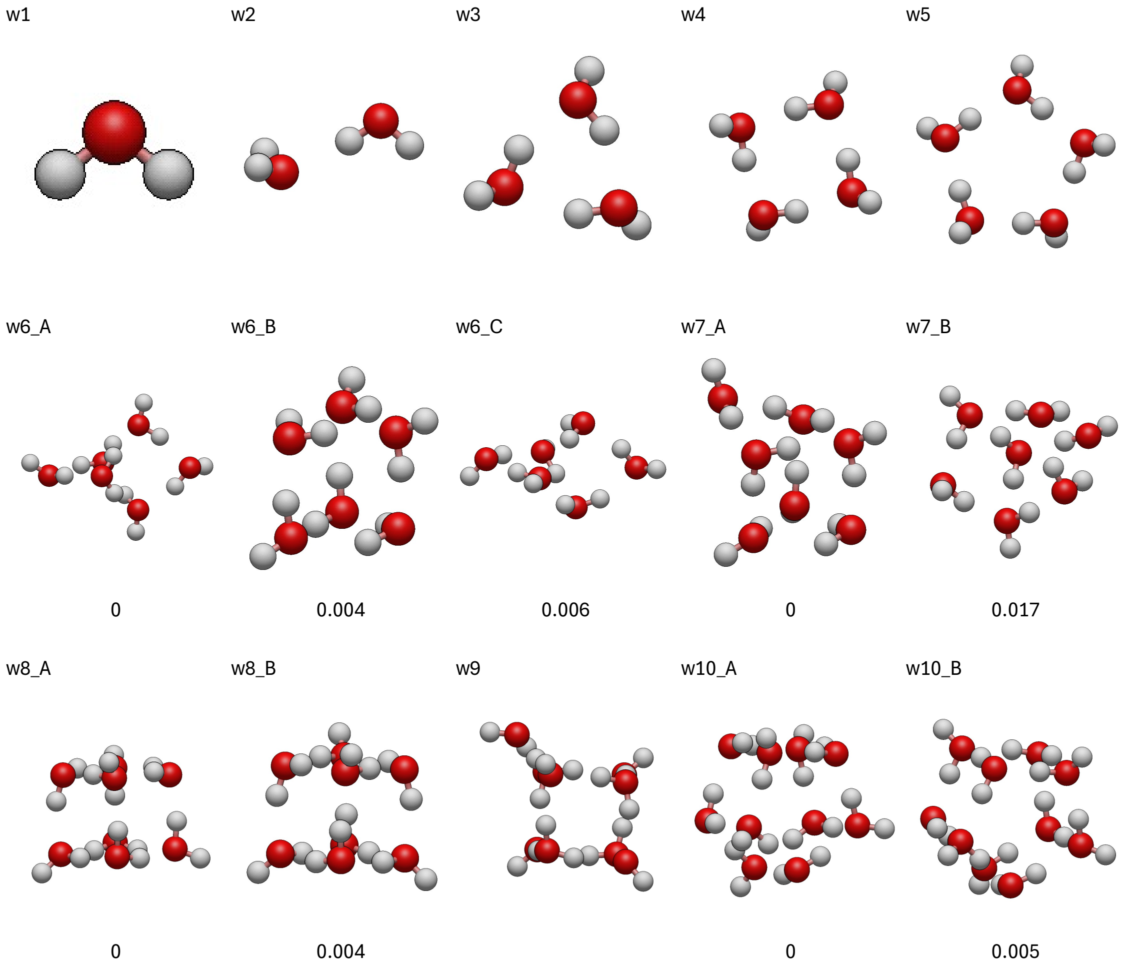

Fig. S15: Isomers of $(H_2O)_{1-10}$ optimized at ωB97XD/aug-cc-pvdz level of theory, energy given below each structure is in eV and includes the zero-point vibrational energy.

Absolute binding strengths and benchmarking

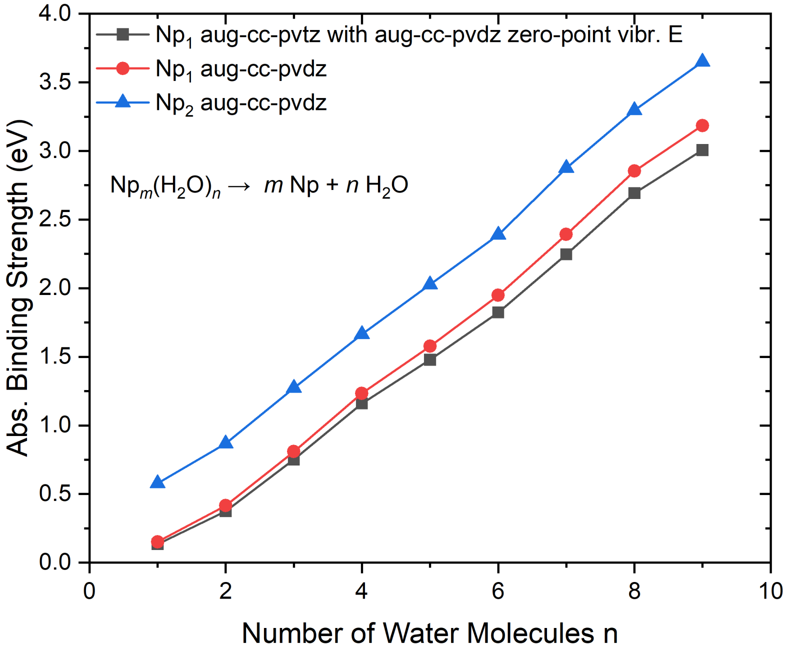

Fig. S16: Absolute binding strength of $(\mathrm{Np})_m(\mathrm{H_2O})_n$ clusters for $m = 1 - 2$ and $n = 1 - 9$. Black squares and red circles correspond to m=1, circles are with energies obtained at ωB97XD/aug-cc-pvdz level of theory including the zero-point vibrational energy, squares represent single-point recalculations in aug-cc-pvtz basis with aug-cc-pvdz zero-point vibrational energy. Blue triangles correspond to m=2 and ωB97XD/aug-cc-pvdz level of theory.

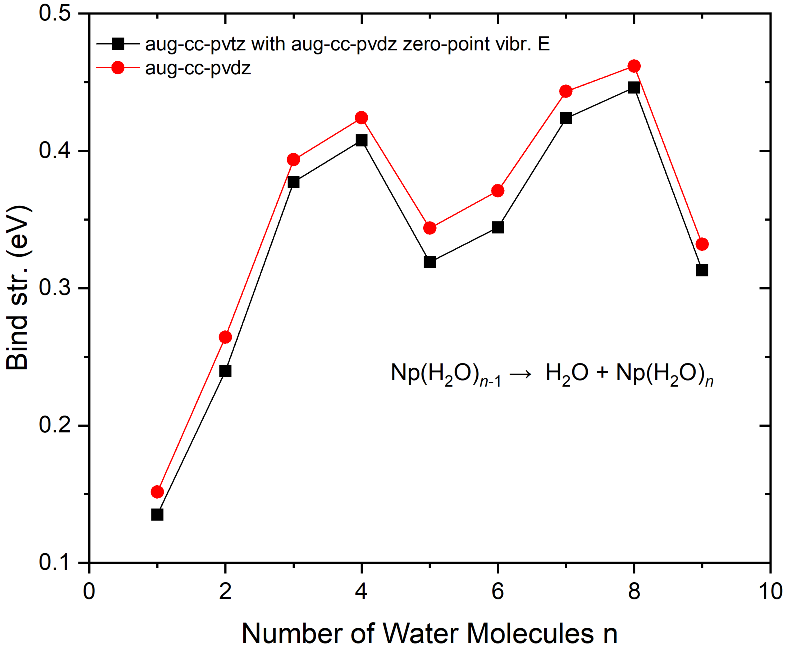

Fig. S17: Incremental binding strength of $(Np)_1(H_2O)_n$ clusters for $n = 1 - 9$. Red circles are calculated with energies obtained at ωB97XD/aug-cc-pvdz level of theory including the zero-point vibrational energy, black squares represent single-point recalculations in aug-cc-pvtz basis with aug-cc-pvdz zero-point vibrational energy.

Binding strength of pure water clusters

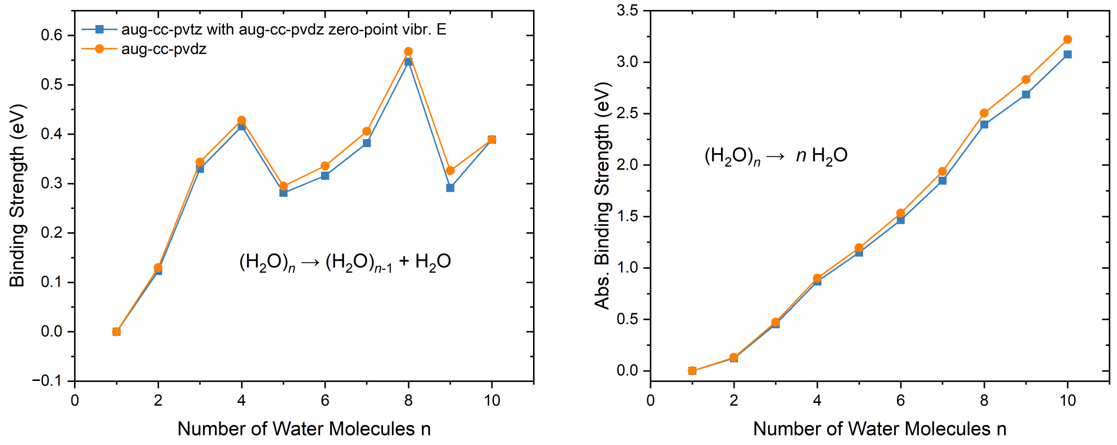

Fig. S18: Incremental (left) and absolute (right) binding strength of pure water clusters evaluated for the process written in each panel. Orange circles correspond to energies obtained at ωB97XD/aug-cc-pvdz level of theory including the zero-point vibrational energy. Blue squares represent single-point recalculations in aug-cc-pvtz basis with aug-cc-pvdz zero-point vibrational energy.

The calculated values of the binding strengths are in good agreement with the benchmark theoretical data[1] or the experiment.[2,3] For example, the result for the water dimer 0.130 eV (ωB97XD/aug-cc-pvdz) compares well with 0.127 eV[1] or 0.137 eV.[2] The absolute binding strength of the trimer corresponds to 0.47 eV in comparison with 0.44 eV,[1] and the incremental binding strength of 0.34 eV agrees well with 0.33 eV in Ref. 3.

Anionic structures

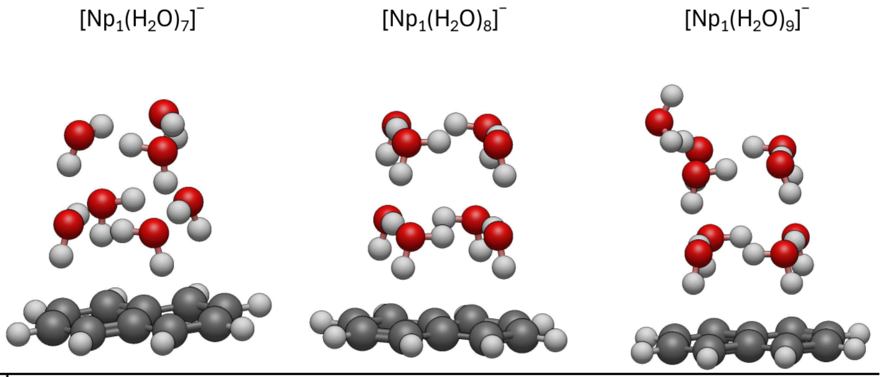

Sum of Mulliken charges on:	n=7	n=8	n=9
$(H_2O)_n$	−0.06	−0.01	0.02
Np	−0.94	−0.99	−1.02

Fig. S19: Structures of anionic $[(\mathrm{Np})_1(\mathrm{H_2O})_{7-9}]^-$ optimized using unrestricted ωB97XD/aug-cc-pvdz method with sums of Mulliken atomic charges on the water cluster and on the naphthalene moiety below each structure.

References

(1) Temelso, B.; Archer, K. A.; Shields, G. C. Benchmark Structures and Binding Energies of Small Water Clusters with Anharmonicity Corrections. *J. Phys. Chem. A* **2011**, *115*, 12034–12046.

(2) Rocher-Casterline, B. E.; Ch'ng, L. C.; Mollner, A. K.; Reisler, H. Determination of the Bond Dissociation Energy (D_0) of the Water Dimer, $(H_2O)_2$, by Velocity Map Imaging. *J. Chem. Phys.* **2011**, *134*, 211101.

(3) Ch'ng, L. C.; Samanta, A. K.; Wang, Y.; Bowman, J. M.; Reisler, H. Experimental and Theoretical Investigations of the Dissociation Energy ($\mathrm{D_0}$) and Dynamics of the Water Trimer, $(H_2O)_3$. *J. Phys. Chem. A* **2013**, *117*, 7207–7216.
